# GLI2 induces genomic instability in human keratinocytes by inhibiting apoptosis

**DOI:** 10.1038/cddis.2013.535

**Published:** 2014-01-30

**Authors:** E Pantazi, E Gemenetzidis, G Trigiante, G Warnes, L Shan, X Mao, M Ikram, M-T Teh, Y-J Lu, M P Philpott

**Affiliations:** 1 Centre for Cutaneous Research, Blizard Institute, Barts and The London School of Medicine and Dentistry, Queen Mary University of London, London, UK; 2 Imaging and Flow Cytometry Core Facilities, Blizard Institute, Barts and The London School of Medicine and Dentistry, Queen Mary University of London, London, UK; 3 Centre for Molecular Oncology, Barts Cancer Institute, Barts and The London School of Medicine and Dentistry, Queen Mary University of London, London, UK; 4 Pathology Core Facilities, Barts Cancer Institute, Barts and The London School of Medicine and Dentistry, Queen Mary University of London, London, UK; 5 Department of Diagnostic and Oral Sciences, Blizard Institute, Barts and The London School of Medicine and Dentistry, Queen Mary University of London, London, UK

**Keywords:** genomic instability, GLI2, BCC, aneuploidy, apoptosis, Bcl-2

## Abstract

Abnormal Sonic Hedgehog signalling leads to increased transcriptional activation of its downstream effector, glioma 2 (GLI2), which is implicated in the pathogenesis of a variety of human cancers. However, the mechanisms underlying the tumorigenic role of GLI2 remain elusive. We demonstrate that overexpression of GLI2-*β* isoform, which lacks the N-terminal repressor domain (GLI2ΔN) in human keratinocytes is sufficient to induce numerical and structural chromosomal aberrations, including tetraploidy/aneuploidy and chromosomal translocations. This is coupled with suppression of cell cycle regulators p21^WAF1/CIP1^ and 14-3-3*σ*, and strong induction of anti-apoptotic signalling, resulting in a reduction in the ability to eliminate genomically abnormal cells. Overexpression of GLI2ΔN also rendered human keratinocytes resistant to UVB-mediated apoptosis, whereas inhibition of B-cell lymphoma 2 (BCL-2) restored endogenous (genomic instability (GIN)) and exogenous (UVB) DNA damage-induced apoptosis. Thus, we propose that ectopic expression of GLI2 profoundly affects the genomic integrity of human epithelial cells and contributes to the survival of progenies with genomic alterations by deregulating cell cycle proteins and disabling the apoptotic mechanisms responsible for their elimination. This study reveals a novel role for GLI2 in promoting GIN, a hallmark of human tumors, and identifies potential mechanisms that may provide new opportunities for the design of novel forms of cancer therapeutic strategies.

Multiple genetic alterations are required for cancer development, and understanding the mechanisms that promote and enhance DNA damage is important. One of the hallmarks of cancer is genomic instability (GIN) and this is reflected in the heterogeneity seen within individual tumors and the extensive genomic alterations in cancer cells, responsible for both cancer development and treatment failure.^1^ GIN refers to a set of events capable of causing unscheduled alterations either of a temporary or permanent nature within the genome.^2^ GIN can be divided into two major groups: (1) microsatellite instability, occurring at the nucleotide level, due to faulty DNA repair pathways and (2) chromosomal instability (CIN), defined as an accelerated rate of chromosomal alterations due to increased chromosome damage, defects in end-joining repair mechanisms and errors in chromosome segregation.^2, 3, 4^ Chromosome alterations, which commonly occur in human cancers, can be classified as structural alterations, including inversions, deletions, amplifications, duplications and translocations, and numerical changes – denoting gains or losses of whole chromosomes, leading to aneuploidy.^2, 4, 5^ Genomic abnormalities tend to induce apoptosis as a default pathway that aborts cells with damaged DNA.^4, 6, 7, 8^ In addition to the increased rate in generating genomic alterations, inactivation of checkpoint and genomic surveillance genes is critical during the development of GIN by allowing the accumulation of gene mutations, chromosome damage and ploidy abnormalities.^2, 3, 4, 7, 8^

Basal cell carcinoma (BCC) of the skin is the most common malignancy in humans. Several studies have provided evidence of GIN in BCCs, highlighting their multiclonal nature and the presence of numerical chromosomal alterations, gene amplification, multinucleated cells and chromosome polysomy.^9, 10, 11, 12, 13, 14, 15^ The development of BCC is associated with aberrant activation of the Hedgehog (HH) signalling pathway, which leads to increased expression and activation of glioma (GLI; GLI1 and GLI2) transcription factors, the principal effectors of the HH pathway.^16^ Targeted expression of an active mutant of GLI2 (GLI2ΔN – a constitutively active form of GLI2 isoform *β*, lacking the N-terminal repressor domain) is sufficient to induce the formation of BCC-like lesions in the epidermis of transgenic mice^17, 18, 19^ and is required for their maintenance.^20^ Moreover, GLI2 is consistently upregulated in tumors that display complex genomic alterations.^21, 22, 23, 24, 25, 26, 27, 28, 29, 30^ However, the role of GLI2 in GIN remains elusive. Therefore, we investigated whether GLI2 influences the genomic integrity of epidermal keratinocytes. We found that ectopic expression of GLI2ΔN profoundly affected the genomic stability of human epidermal keratinocytes, partly by suppressing cell cycle checkpoint proteins and disabling the intrinsic apoptotic mechanisms responsible for the elimination of cells with genomic alterations, thus identifying a novel role for GLI2 in tumorigenesis.

## Results

### Generation and characterisation of EGFP-GLI2ΔN stable expressing cells

Human telomerase-immortalised newborn epidermal (N/TERT) keratinocytes stably expressing enhanced green fluorescent protein (EGFP; SINCE) or enhanced green fluorescent protein-GLI2-*β* isoform, which lacks the N-terminal repressor domain (EGFP-GLI2ΔN; SINEG2) were generated and expression of the fusion product was confirmed by mRNA expression, fluorescent microscopy, flow cytometry and western blot analysis (Supplementary Figure S1). The effect of GLI2ΔN on N/TERT cell proliferation was analysed by the Alamar blue and MTT (3-(4,5-dimethylthiazol-2-yl)-2,5-diphenyl tetrazolium bromide) cell viability assays, revealing significantly less SINEG2 cells compared with parental N/TERT or N/TERT keratinocytes stably expressing EGFP (SINCE) cells after 7 days in culture. Over prolonged culture (16 days), SINEG2 cells underwent fewer population doublings than either of the control cells. Collectively, these data show that ectopic GLI2ΔN reduces the proliferation rate of N/TERT cells (Supplementary Figure S2).

### GLI2 induces tetraploidy and numerical chromosomal alterations

**Figure 1:**
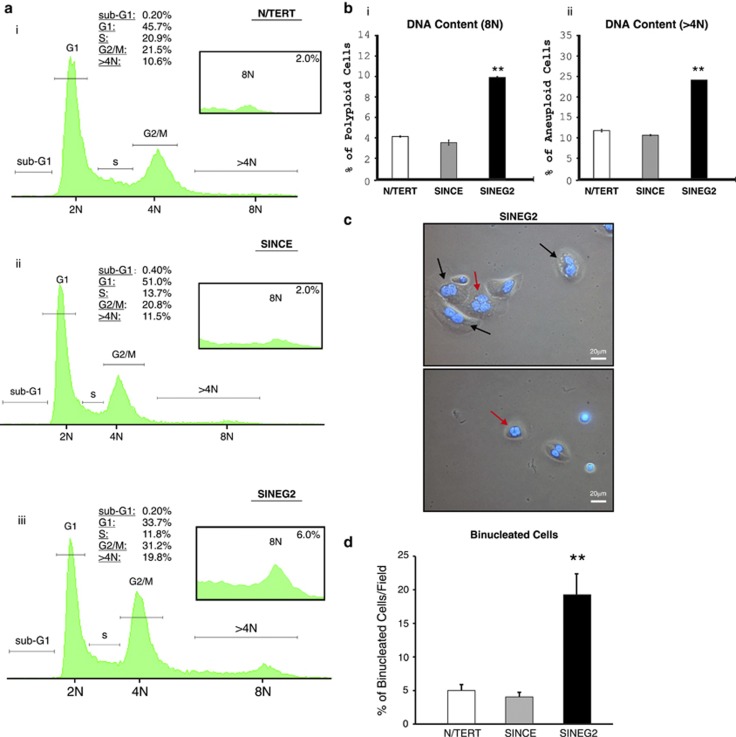
GLI2ΔN induces tetraploidy, polyploidy and aneuploidy in N/TERT keratinocytes. (**a**) Propidium iodide staining, followed by flow cytometry analysis to obtain cell cycle distribution of N/TERT (i), SINCE (ii) and SINEG2 (iii) cells. Sub-G1 trace was negligible for all cell lines examined. Data are representative of three independent experiments. (**b**) Graphical representation of the percentage of (i) 8N and (ii) >4N cells for each cell line after Hoechst-33342 staining and flow cytometry analysis. SINEG2 cells have significantly higher *(P*≤0.01) percentage of polyploid and aneuploid cells in culture. Each bar represents the mean values±S.E.M. of duplicate samples. (**c**) Representative pictures taken from Hoechst-33342-stained SINEG2 cell line at × 20 magnification, showing the presence of bi- and multinucleated cells. Black arrows indicate binucleated cells and red arrows indicate multinucleated cells. DNA was detected by Hoechst-33342 staining (blue). (**d**) N/TERT, SINCE and SINEG2 cells were stained with DAPI to visualise nuclear DNA. SINEG2 cells display a significant increase (∼3.5 fold) in the population of binucleated (tetraploid/near-tetraploid) cells. Each bar represents the mean values of binucleated cells per field±S.E.M. of 7 fields for N/TERT and SINCE, and 13 fields for SINEG2. Total count ∼300 cells per cell line. **P≤0.01. N, haploid number

Cell cycle analysis after Hoescht-33342 staining revealed a significant increase in the 4N population in SINEG2 (Supplementary Figure S3), which could be caused either by a G2/M block, or by an abnormal accumulation of tetraploid/near-tetraploid cells. The latter was confirmed by further analysis using propidium iodide, which showed that SINEG2 cells have a significant increase in the percentage of polyploid and aneuploid cells with 8N and >4N, compared with N/TERT and SINCE cells (Figures 1a and b), indicating that GLI2ΔN expression promotes polyploidy and aneuploidy. Similarly, cell cycle analysis in primary normal human epidermal keratinocytes (NHEKs) and in human uterus endometrium leiomyosarcoma (SK-UT-1B) diploid cells, overexpressing GLI2ΔN, showed a significant increase in the percentage of 4N and >4N cells (Supplementary Figure S4). We also found enlarged, bi- and multinucleated SINEG2 cells by *in situ* Hoechst-33342 staining (Figure 1c), indicating the existence of binucleated tetraploid/near-tetraploid and multinucleated polyploid and aneuploid cells, caused by cytokinesis failure.

Furthermore, we counted the proportion of binucleated N/TERT, SINCE and SINEG2 cells stained with DAPI and found a significantly *(P<0.01)* elevated percentage of binucleated cells (∼19%) in SINEG2 compared with both control cell lines (5.4% for N/TERT and 4.2% for SINCE; Figure 1d). The difference in binucleated cells (∼14%) is consistent with the differences in 4N populations measured by flow cytometry (∼11–15%) between control (N/TERT and SINCE) and SINEG2 keratinocytes (Supplementary Figure S3 and Figure 1a), suggesting that the accumulation of 4N SINEG2 cells, observed by flow cytometry, is mainly due to the presence of tetraploid/near-tetraploid cells rather than the activation of the G2/M checkpoint of diploid cells. This is further supported by the 8N and >4N DNA content cells (Figures 1a and c). However, a transient arrest of cells, due to activation of the mitotic spindle checkpoint, cannot be excluded completely.

### GLI2 induces structural chromosomal abnormalities

**Figure 2:**
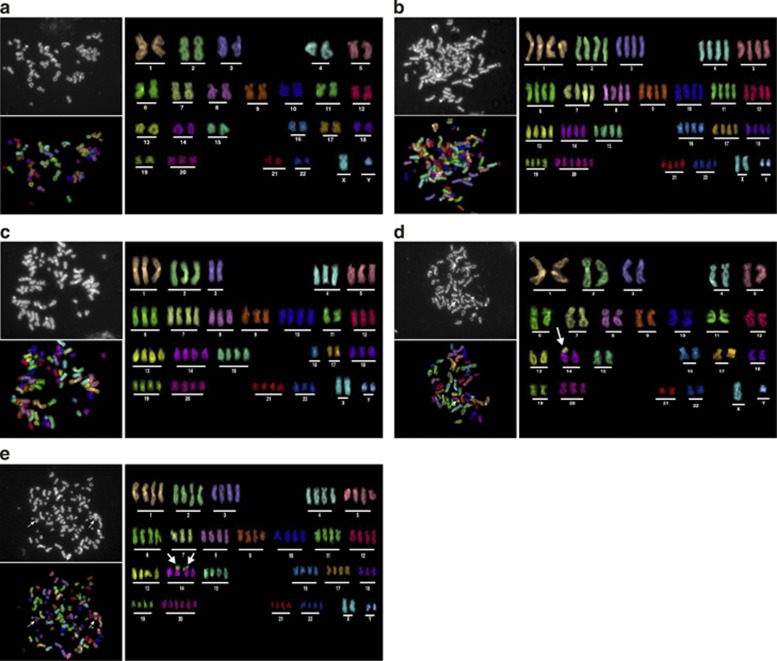
GLI2ΔN induces numerical and structural chromosomal changes in human keratinocytes. (**a**) Representative metaphase cell from SINCE as a DAPI-counterstained image (upper left), with the 24-colour-painted chromosomes (lower left) and a karyotype of 47, XY, +20, based on the colour code (right). (**b**) Representative tetraploid metaphase cell from SINEG2 as a DAPI-counterstained image (upper left), with the 24-colour-painted chromosomes (lower left) and a karyotype of 94, XXYY, +20 × 2 based on the colour code (right). (**c**) Metaphase aneuploid (near-tetraploid aneuploid) SINEG2 cell as a DAPI-counterstained image (upper left), with the 24-colour-painted chromosomes (lower left) and a karyotype of 73, XXYY, −1, −2, −3 × 2, −4, −5, −6, −8, −9, −11 × 2, −12, −16 × 3, −17 × 2, −18 and −22, based on the colour code (right). This is an example of a tetraploid cell that might have undergone multipolar and asymmetric divisions, yielding aneuploid progenies. (**d**) Diploid SINEG2 metaphase cell with the clonal nonreciprocal unbalanced translocation t(7;14; white arrows) as a DAPI-counterstained image (upper left), with the 24-colour-painted chromosomes (lower left) and a karyotype of 47, XY, −7, +del7q, −14, +der(14)t(7;14), +20, based on the colour code (right). (**e**) Representative near-tetraploid SINEG2 metaphase cell with the clonal nonreciprocal unbalanced translocation t(7;14; white arrows; upper left), with the 24-colour-painted chromosomes (lower left) and a karyotype of 92, XXYY, −7 × 2, +del7q, −14 × 2, +der(14)t(7;14)X2, −18, +20 × 2, based on the colour code (right)

We also revealed structural chromosomal abnormalities in GLI2ΔN-expressing keratinocytes. Multiplex fluorescent *in situ* hybridisation (M-FISH) analysis revealed a stable karyotype of 47,XY,+20, therefore with the presence of an extra chromosome 20 (trisomy 20) in the near-diploid male, accounting for ∼90% of metaphases analysed from keratinocyte cell lines N/TERT and SINCE (Figure 2a). The rest were tetraploid cells with double number of each chromosome in the near-diploid cells. Trisomy 20 was further confirmed by 10 K SNP (single nucleotide polymorphism) array analyses (GEO accession number: GSE36105), using normal donor human skin keratinocytes as reference cells (Supplementary Figure S5). No structural chromosome aberrations were detected in the control N/TERT and SINCE cells (Figure 2).

However, SINEG2 keratinocytes showed both numerical and structural chromosome aberrations (Figure 2). Approximately 14% of metaphase SINEG2 cells were tetraploid (94 chromosomes with the karyotype 94, XXYY, +20 × 2; Figure 2b) and∼70% were near-tetraploid/aneuploid cells with random gains and losses of chromosomes (i.e., 73 chromosomes; Figure 2c), in contrast to control cells of which only ∼10% were tetraploid. Interestingly, we found structural chromosomal rearrangements in ∼29% (4/14) karyotyped metaphases of SINEG2 keratinocytes, including a clonal non-reciprocal chromosome translocation; t(7;14) in 3 of 14 karyotyped metaphases (Figures 2d and e), 1 is diploid (Figure 2d) and the other 2 are near-tetraploid/aneuploid cells (Figure 2e). A non-clonal unbalanced translocation t(9;14) was also detected in a near-tetraploid/aneuploid SINEG2 keratinocyte (data not shown).

### GLI2ΔN induction leads to impaired checkpoint control and apoptosis for the survival of cells with chromosomal abnormalities

Cells with numerical or structural chromosome changes usually stimulate cell cycle p53/p21^WAF1/CIP1^-dependent checkpoint arrest and/or subsequent apoptosis.^4, 7, 8, 27, 31, 32, 33, 34, 35, 36^ To understand how such populations are maintained, we investigated whether, along with the generation of cells with accumulated genomic alterations, GLI2ΔN also prevents the induction of the ‘tetraploidy checkpoint' and apoptosis.

**Figure 3:**
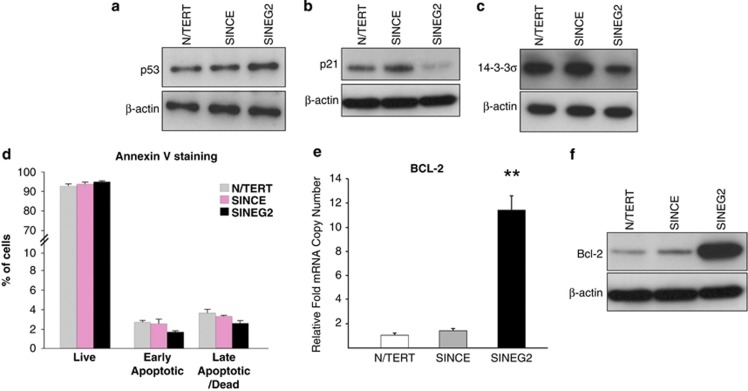
GLI2ΔN expression abolishes cell cycle checkpoints and induces anti-apoptotic signalling. SINEG2 cells show a marginal increase of p53 protein (**a**), a significant decrease of the CDK inhibitor p21^WAF1/CIP1^ protein (**b**) and a marked decrease in the 14-3-3*σ* protein levels (**c**), compared with both N/TERT and SINCE control cells. *β*-Actin was used as a protein-loading control. (**d**) N/TERT, SINCE and SINEG2 cells stained with Cy5 Annexin V and DAPI for the detection of apoptotic and dead cells, respectively. The percentage of cells representing the early (Annexin V (+) DAPI (−)) and late (Annexin V (+) DAPI (+)) apoptotic or dead populations show no significant differences. Each bar represents the mean values±S.E.M. of three independent experiments, each of which consisted of duplicate samples. BCL-2 is upregulated in SINEG2 cells both at RNA (**e**) and protein (**f**) level, compared with N/TERT and SINCE control cells. In **e**, each bar represents mean fold induction/or suppression relative to N/TERT (arbitrary value of 1)±S.E.M. of triplicate samples. **P≤0.01

Although we detected a marginal increase of p53 protein levels in SINEG2 keratinocytes (Figure 3a), its direct transcriptional target p21^WAF1/CIP1^ was significantly downregulated (Figure 3b), indicating a defective G1 ‘tetraploidy checkpoint' in GLI2ΔN-expressing cells. This demonstrates the ability of GLI2ΔN to suppress p21^WAF1/CIP1^ in a p53-independent manner. p21^WAF1/CIP1^ was also found to be suppressed in human BCCs, which are GLI2-driven tumors sharing many attributes with the GLI2ΔN-expressing keratinocytes (Supplementary Figure S6). 14-3-3*σ*, another direct transcriptional target of p53 after DNA damage and lost in BCCs,^37^ was also downregulated in SINEG2 compared with both N/TERT and SINCE keratinocytes (Figure 3c).

Following Annexin V staining, flow cytometry analysis showed that upon expression of GLI2ΔN, despite the presence of GIN, there was no difference in numbers of live, early apoptotic, late apoptotic/dead cells, between control and SINEG2 cells (Figure 3d), indicating lack of apoptosis induced by genomic changes in GLI2ΔN-expressing cells.

B-cell lymphoma 2 (BCL-2) is a direct target of GLI2ΔN in HaCaT keratinocytes and is highly expressed in human BCCs.^38^ We found that the level of BCL-2 mRNA was elevated by 11-fold in SINEG2 compared with N/TERT and SINCE controls (*P*≤0.01; Figure 3e), whereas Bcl-2 protein (Figure 3f) was massively upregulated in SINEG2 cells, suggesting a role in preventing apoptosis in SINEG2 cells.

**Figure 4:**
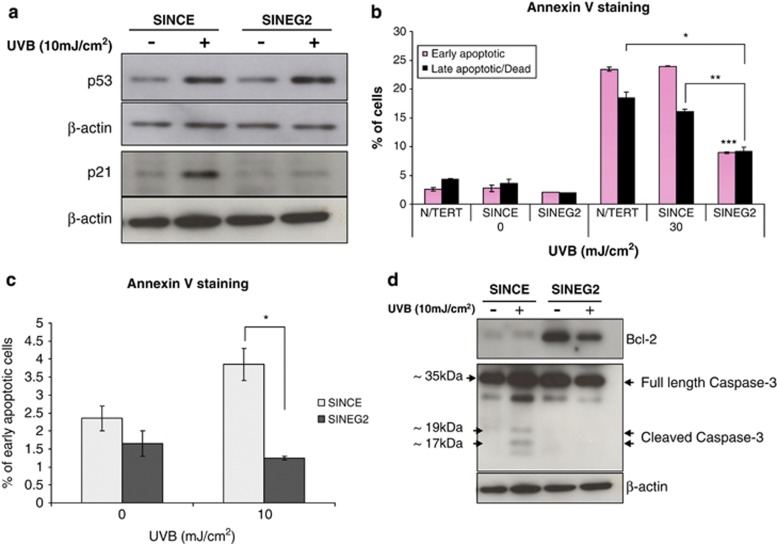
GLI2ΔN-expressing keratinocytes escape the UVB DNA damage response by p53-independent downregulation of p21 and display resistance to UVB-mediated apoptosis. (**a**) Immunoblotting analysis for p53 and p21 on UV (+) and non-UV (−) SINCE and SINEG2 whole-cell lysates. p53 is upregulated in SINCE control and SINEG2 cells on UVB exposure, whereas p21 is only increased in control cells but not in GLI2ΔN-expressing cells after UVB. (**b**) N/TERT, SINCE and SINEG2 keratinocytes either mock treated (0 mJ/cm^2^) or UVB irradiated with 30 mJ/cm^2^. SINEG2 keratinocytes display a reduction in the percentage of early and late apoptotic/dead populations in response to high dose of UVB irradiation compared with both N/TERT and SINCE control keratinocytes. Each bar represents the mean values±S.E.M. of duplicate samples. *P≤0.05, **P≤0.01, ***P≤0.001. (**c**) SINCE and SINEG2 keratinocytes, either mock (0 mJ/cm^2^) or UVB (10 mJ/cm^2^) treated. It shows that SINEG2 cells are less susceptible to UVB-induced early apoptosis compared with SINCE control cells. Each bar represents mean values±S.E.M. of duplicate samples. *P≤0.05. (**d**) SINCE and SINEG2 keratinocytes were collected for total protein, 24 h after UVB 10 mJ/cm^2^ (+) or mock (−) 0 mJ/cm^2^ treatment. Total protein extracts were immunoblotted against anti-Bcl-2 (∼26 kDa; top panel) and anti-full length (∼35 kDa), and cleaved Caspase-3 (∼19, 17 kDa; middle panel) antibodies. *β*-Actin (∼42 kDa) was used as a protein-loading control. The absence of activated (cleaved) Caspase-3 in SINEG2 cells, a late apoptotic marker, following UVB irradiation, confirms the apoptotic resistance of SINEG2 keratinocytes compared with SINCE cells

As GLI2ΔN-expressing cells are resistant to tetraploidy-mediated apoptosis, we investigated whether GLI2 overexpression can also render cells resistant to other apoptotic stimuli such as UV light, which is known to cause p53-mediated induction of p21^WAF1/CIP1^ and apoptosis. UVB irradiation is an established risk and aetiological factor in human BCC formation. Following UVB irradiation, p53 was upregulated in both GLI2ΔN-expressing and control keratinocytes (Figure 4a). However, p21^WAF1/CIP1^ was upregulated only in control cells (Figure 4a) but remained suppressed in SINEG2 cells (Figure 4a), further confirming that GLI2 suppresses p21 expression independently of p53. The apoptotic response of N/TERT, SINCE and SINEG2 cells following UVB irradiation (10 or 30 mJ/cm^2^) was measured against mock-irradiated controls (0 mJ/cm^2^). SINEG2 display a reduction of early apoptotic and late apoptotic/dead cells in response to high (30 mJ/cm^2^) dose of UVB irradiation (∼9% early apoptotic cells), compared with both N/TERT (∼24% early apoptotic cells) and SINCE (∼24% early apoptotic cells) control keratinocytes (Figure 4b). Similar results were obtained with a lower (10 mJ/cm^2^) UVB dose *(P*<0.001; Figure 4c). Furthermore, the absence of activated/cleaved Caspase-3 (Figure 4d), in combination with the high-level of Bcl-2 (Figure 4d), further confirm that SINEG2 keratinocytes are able to escape p53-mediated apoptosis.

### Inhibition of BCL-2 restores endogenous (chromosomal alterations) and exogenous (UVB) DNA-damage-induced apoptosis to GLI2ΔN-expressing keratinocytes

**Figure 5:**
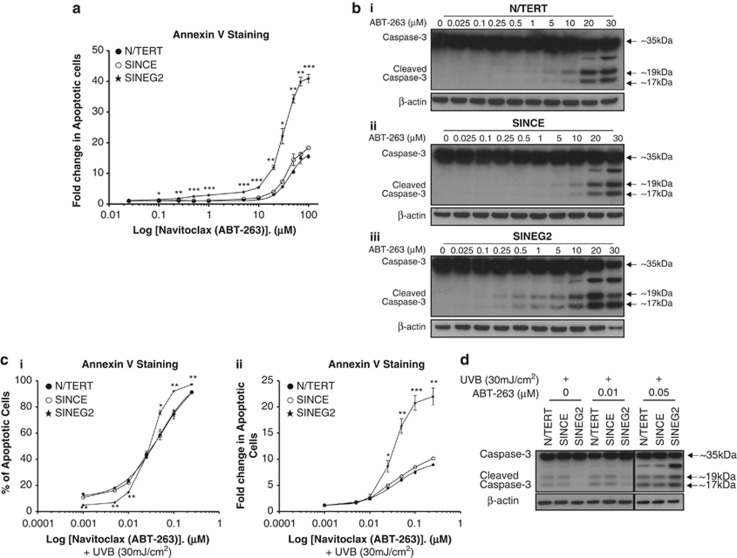
BCL-2 inhibition leads to apoptosis sensitisation of GLI2ΔN-expressing cells and abolishes their resistance to UVB-mediated apoptosis. (**a**) N/TERT, SINCE and SINEG2 cells were either untreated (0 *μ*M/DMSO) or treated with increasing concentrations of Navitoclax (ABT-263; 0.025–100 *μ*M) and were incubated for 24 h. Next, apoptosis was detected by Cy5 Annexin V and DAPI staining, followed by flow cytometry analysis showing that SINEG2 cells have much higher apoptotic sensitivity to BCL-2 inhibition compared with control cells. Each point represents the mean fold change in the number of early (Annexin V (+) DAPI (−)) and late (Annexin V (+) DAPI (+)) apoptotic cells relative to the treatment with vehicle-only (0 *μ*M/DMSO) control for each individual cell line (arbitary value 1)±S.E.M. of duplicate samples. *P≤0.05, **P≤0.01, ***P≤0.001. (**b**(i)) N/TERT, (ii) SINCE and (iii) SINEG2 cells were either untreated (0 *μ*M/DMSO) or treated with increasing doses of Navitoclax (ABT-263; 0.025–30 *μ*M) and were incubated for 24 h. Next, cells were collected and whole-cell lysates were prepared and immunoblotted against anti-full-length Caspase 3 (∼35 kDa) antibody, whereas *β*-actin (∼42 kDa) was used as a protein-loading control. Apoptosis was assessed through detecting the cleaved bands of Caspase-3 (∼19, 17 kDa). In **c** and **d**, N/TERT, SINCE and SINEG2 cells were either untreated (0 *μ*M/DMSO) or treated with different concentrations of Navitoclax (ABT-263; 0.001–0.25 *μ*M) for up to 24 h. Cells were irradiated with a single dose of 30 mJ/cm^2^ and were allowed to grow for another ∼20 h. Next, apoptosis was detected either by (**c**) Cy5 Annexin V and DAPI staining, followed by flow cytometry analysis or by (**d**) immunoblotting analysis, showing that inhibition of BCL-2 abolishes the resistance of GLI2ΔN-expressing cells to UVB-induced apoptosis. (**c**(i)) Each point represents the mean values/percentages of early (Annexin V (+) DAPI (−)) and late (Annexin V (+) DAPI (+)) apoptotic cells±S.E.M. of three independent experiments. *P≤0.05, **P≤0.01; (ii) each point represents the mean fold change in the number of early (Annexin V (+) DAPI (−)) and late (Annexin V (+) DAPI (+)) apoptotic cells relative to the treatment with vehicle-only (0 *μ*M/DMSO) and 30 mJ/cm^2^ UVB control for each individual cell line (arbitary value 1)±S.E.M. of three independent experiments. *P≤0.05, **P≤0.01, ***P≤0.001. (**d**) Whole-cell lysates were prepared and immunoblotted against anti-full-length Caspase 3 (∼35 kDa) antibody, whereas *β*-actin (∼42 kDa) was used as a protein-loading control

Activation of the anti-apoptotic signalling is essential for the survival of cells with endogenous (i.e., GIN) or exogenous (i.e., UVB) DNA damage, and is one of the mechanisms employed by cancer cells to evade apoptosis. Our results suggest a pivotal role for BCL-2 in the survival of cells overexpressing GLI2ΔN. We inhibited BCL-2 in N/TERT, SINCE and SINEG2 keratinocytes using a range of concentrations of Navitoclax (ABT-263), and measured apoptosis by Annexin V staining and cleavage of Caspase-3. Navitoclax is a potent inhibitor of the Bcl-2/Bcl-xL proteins, which restores the intrinsic pathway activity in response to apoptotic stimuli.^39, 40^ By Annexin V flow cytometry analysis, we show that SINEG2 cells become highly sensitised to low and high doses of Navitoclax (Figure 5a and Supplementary Figure S7A), which correlates with the increased cleavage of Caspase-3 protein (Figure 5b (iii) and Supplementary Figure S7B), which occurred even at the low dose of 0.1 *μ*M of Navitoclax (Figure 5b (iii)). In contrast, normal N/TERT and SINCE control cells required at least a minimum dose of 5 *μ*M before Caspase-3 protein cleavage becomes detectable (Figure 5b (i and ii) and Supplementary Figure S7B), suggesting lower apoptotic induction of normal human keratinocytes to BCL-2 inhibition compared with GLI2ΔN-expressing keratinocytes.

To determine whether the resistance of GLI2ΔN-expressing cells to UVB-induced apoptosis can be attributed to the elevated levels of BCL-2, we investigated the effects of Navitoclax on apoptosis of SINEG2 cells in response to UVB (30 mJ/cm^2^). Much lower doses of Navitoclax (ABT-263; 0.001–0.25 *μ*M) were used due to possible synergistic effects with UVB. Annexin V staining (Figure 5c (i)) and immunoblotting analysis (Figure 5d and Supplementary Figure S7C) showed that SINEG2 cells retain their resistance to UVB-induced apoptosis, during treatment with very low doses of Navitoclax (0.001–0.01 *μ*M), compared with N/TERT and SINCE control cells. These doses of the drug may be insufficient to fully inhibit the function of BCL-2, especially in SINEG2 cells where the basal levels of BCL-2 protein are dramatically elevated. Consistently, at higher doses of Navitoclax (>0.01 *μ*M), the number of apoptotic SINEG2 cells rises sharply and surpasses that of N/TERT and SINCE cells (0.05–0.25 *μ*M; Figure 5c), again indicating increased induction of apoptotic cell death in SINEG2 cells. Immunoblotting analysis showed that the cleavage of Caspase-3 at doses >0.01 *μ*M (i.e., 0.05 *μ*M) was higher in SINEG2 than in N/TERT and SINCE control cells (Figure 5d and Supplementary Figure S7C).

**Figure 6:**
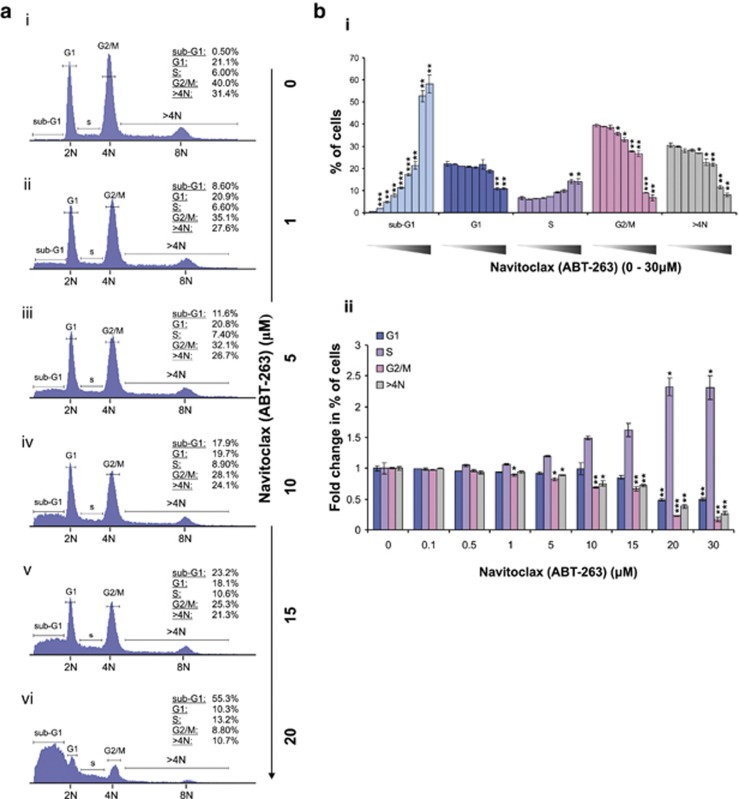
BCL-2 inhibition leads to the induction of apoptosis and reduction of abnormal tetraploid and aneuploid cells in SINEG2 cultures. SINEG2 cells were either untreated (0 *μ*M/DMSO) or treated with increasing concentrations of Navitoclax (ABT-263; 0.1–30 *μ*M) and were incubated for 24 h. Next, cells were stained with propidium iodide (PI) followed by flow cytometry analysis to obtain cell cycle distribution of SINEG2 cells. (**a**) DNA content profiles of SINEG2 cells after treatment with various doses of Navitoclax (ABT-263) (i–vi). Data are representative of three independent experiments, each of which consisted of duplicate samples. N, haploid number. (**b**) Graphical representations of flow cytometry analysis; (i) each bar represents the mean values/percentages of SINEG2 cells±S.E.M. of duplicate samples present in each different phase of the cell cycle (apoptotic sub-G1, G1, S, G2/M, DNA content >4N). *P≤0.05, **P≤0.01, ***P≤0.001. Black arrow represents the increasing doses of Navitoclax (ABT-263; 0/DMSO, 0.1, 0.5, 1, 5, 10, 15, 20, 30 *μ*M); (ii) each bar represents the mean fold change in the number of treated SINEG2 cells relative to the treatment with vehicle-only (0 *μ*M/DMSO) control for each different phase of the cell cycle (G1, S, G2/M, DNA content >4N; arbitary value 1)±S.E.M. of duplicate samples. *P≤0.05, **P≤0.01, ***P≤0.001

Our results indicate that the survival of GLI2ΔN-expressing cells largely depends on BCL-2. Once its function is pharmacologically inhibited, cells become hyper-sensitised to apoptotic cell death. As tetraploidy/aneuploidy itself is a strong apoptotic signal, blocking the function of BCL-2 is expected to induce a marked apoptotic response and the reduction of abnormal tetraploid/aneuploid progenies. We tested this hypothesis by treating SINEG2 cells with a range of doses of BCL-2 inhibitor Navitoclax (ABT-263) before examining their DNA content profile. As expected, treatment over a range of concentrations of Navitoclax induced much higher levels of apoptosis in SINEG2 cells, detectable by DNA fragmentation (sub-G1; Figures 6a and b (i)), compared with normal control N/TERT and SINCE cells (Supplementary Figure S7D). The numbers of cells with DNA content 4N (G2/M normal diploid cells and tetraploid/near-tetraploid G1 cells), as well as those with DNA content >4N (polyploid and aneuploid cells), also decreased with increasing concentrations of Navitoclax. By quantifying the numbers of cells in each group, we observed that cells with DNA content of 4N and those with DNA content >4N were most significantly affected by treatment with Navitoclax (Figure 6b).

The fact that N/TERT and SINCE cells are not equally sensitised to BCL-2 inhibition compared with SINEG2 cells suggests that the tetraploid and near-tetraploid G1 SINEG2 cells (4N) may be more sensitive to BCL-2 inhibition than the diploid G2/M cells (also 4N), due to apoptosis induced by genomic changes. Interestingly, the number of S-phase cells is also gradually elevated with increasing doses of the drug in SINEG2 cells (Figure 6b) compared with that in N/TERT and SINCE normal diploid control cells (Supplementary Figure S7D). This population represents cells with a possible S-phase arrest and/or 4N cells with fragmented DNA due to apoptosis. As the normal control N/TERT and SINCE cells do not show any evidence of S-phase arrest (Supplementary Figure S7D), the increased number of SINEG2 cells in S phase may consist mainly of apoptotic tetraploid and near-tetraploid G1 cells (4N). The number of cells with DNA content 2N (G1) appears to be stable across treatment with different doses of Navitoclax and only shows a significant decrease at 20 and 30 *μ*M of Navitoclax (Figure 6b). However, this reduction in cell number is less than that seen for cells with DNA content 4N and >4N. Collectively, we showed that inhibition of BCL-2 function by Navitoclax affects the survival of genomically unstable tetraploid/aneuploid SINEG2 cells more than stable diploid cells.

## Discussion

GLI2 is frequently overexpressed in human cancers associated with aneuploidy and CIN, including human BCCs, prostate, breast, hepatocellular, colon and oral squamous cancers, osteosarcomas and melanomas.^5, 10, 12, 21, 22, 23, 24, 25, 26, 27, 28, 29, 30, 38, 41, 42, 43, 44^ However, its role in aneuploidy and CIN has not previously been reported. In this study we showed that stable overexpression of the constitutively active form of GLI2 in N/TERT human epidermal keratinocytes resulted in the accumulation and increased viability of cells with both numerical and structural chromosomal alterations, revealing potential novel mechanisms by which GLI2 might exert its tumorigenic effect.

Cell cycle analysis of N/TERT keratinocytes expressing GLI2ΔN showed that cells displayed an increased percentage of 4N cells, including many binucleated tetraploid cells of G1 biochemical state, as well as an increased proportion of polyploid and aneuploid cells with DNA content 8N and >4N representing G2/M- and S-phase tetraploid/near-tetraploid aneuploid cells. These findings indicate that overexpression of GLI2ΔN can interfere with normal cell cycle completion. This effect is not cell line specific, as we observed similar effects when GLI2ΔN was overexpressed in NHEK and in the diploid tumorigenic line SK-UT-1B. Moreover, the increase in tetraploid cells observed on GLI2ΔN induction may be a contributing factor in cell transformation and carcinogenesis, by promoting GIN/CIN and the formation of aneuploid cells.^6, 33^ Tetraploidy is commonly seen in human cancer, including GLI2ΔN-driven malignancies, such as BCCs,^5, 6, 27, 45, 46, 47^ and has been reported as an early step in human tumorigenesis and a contributing factor in the generation of aneuploid tumors.^4, 6, 7, 27, 33, 48, 49, 50^ Tetraploidy also has its own tumorigenic potential, as only tetraploid, and not diploid, p53-null mouse mammary epithelial cells promote tumorigenesis after subcutaneous injection into nude mice.^48^

In addition to numerical chromosomal aberrations, GLI2ΔN-overexpressing keratinocytes presented with structural chromosomal abnormalities, such as translocations. The identification of the clonal translocation t(7;14) both in near-tetraploid/aneuploid and diploid SINEG2 keratinocytes on GLI2ΔN induction suggests that this chromosome translocation event occurs in the diploid cell and thus the ability of GLI2ΔN to induce structural chromosomal aberrations is independent of the induction of tetraploidisation.

Here we propose two potential mechanisms by which GLI2ΔN is able to induce tetraploidisation. First, by indirectly downregulating 14-3-3*σ*, whose loss leads to the improper mitotic cap-independent translation of important executioners of mitosis, cytokinesis failure and accumulation of binucleate tetraploid cells,^51^ and is frequently seen in many cancers, including breast, prostate and BCCs.^37, 52^ Second, the downregulation of p21^WAF1/CIP1^ upon GLI2ΔN induction *in vitro*, which was confirmed in human BCCs *in vivo*, might be another mechanism to promote tetraploidisation. As p21^WAF1/CIP1^ affects centrosome homeostasis, and its deficiency triggers a *bona fide* overduplication of centrioles and thus aberrant centrosome numbers,^53^ loss of p21^WAF1/CIP1^ may induce centrosome overduplication-associated cytokinesis failure,^54^ resulting in increased binucleated tetraploidy and genomic doubling.

Importantly, a low but detectable population of cells with 8N DNA content was present in N/TERT and SINCE control cells. This suggests the presence of spontaneously arising tetraploid cells within the normal population. This is consistent with a report showing that human N/TERT keratinocytes have low rates of chromosomal non-disjunction during normal bipolar mitoses, which leads to the generation of binucleated tetraploid progenies (∼2%) due to cytokinesis failure, instead of aneuploid progenies.^6, 46^ However, the majority of N/TERT binucleated tetraploids did not proceed to the next division,^46^ possibly due to the activation of p53/p21^WAF1/CIP1^-dependent checkpoint followed by apoptosis, previously shown to be responsible for the restricted growth potential of tetraploid cells.^4, 7, 8, 27, 31, 32, 33, 34, 35, 36^ Consistently, a recent study^55^ showed that elevating chromosome missegregation rates alone in human cultured cells is not sufficient to convert stable, near-diploid cells into highly aneuploid cells with karyotypes resembling tumor cells. Thus, activation of oncogenes and/or loss of tumor suppressor genes might be necessary at a subsequent step, enabling binucleated tetraploid cells to survive, proliferate and potentially give rise to aneuploid cells. Taking into account the pivotal role of p21^WAF1/CIP1^ and 14-3-3*σ* in the maintenance of genomic integrity of cells^2, 3, 8, 56^ and the prevention of tetraploidisation and polyploidisation,^4, 7, 51, 53, 57, 58, 59^ the loss of both in GLI2ΔN-expressing keratinocytes suggests a possible contribution not only in the production of binucleated tetraploid cells but also in the progression of cells with structural/numerical chromosomal abnormalities through G1/S and G2/M cell cycle phases, yielding polyploid and aneuploid progenies.

However, similar to the suppression of cell cycle regulators, the inhibition of tetraploidy-induced apoptosis by GLI2 overexpression may be critical in the generation of GIN. It is known that overexpressed BCL-2 inhibits apoptosis after a failed cell division, resulting in enhanced survival of tetraploid cells, which frequently undergo multipolar and asymmetric divisions, leading to aneuploidy and extensive chromosomal alterations.^4, 7, 31, 60^ We found that GLI2ΔN induced BCL-2 overexpression, and by pharmacological inhibition of BCL-2 we confirmed that in GLI2ΔN-expressing keratinocytes, the anti-apoptotic function of BCL-2 is more important to those tetraploid/aneuploid cells than the genomically unchanged cells. This not only showed that loss of BCL-2 function can restore the apoptotic mechanism in GLI2ΔN-expressing cells but also that genomically unstable cells rely much more on the apoptosis-prevention role of BCL-2 than genomically stable cells. In addition, by inhibiting the function of BCL-2 protein by Navitoclax we showed that increased basal levels of BCL-2 are responsible for the resistance of GLI2ΔN keratinocytes to UVB-mediated apoptosis. This is consistent with the apoptotic resistance, mediated by intrinsic or extrinsic pathways, in human BCCs.^38, 61, 62^ Overall, GLI2ΔN-expressing keratinocytes are resistant to both chromosomal alteration-mediated and UVB/DNA-damage-induced apoptosis, and this may explain how cells with genomic aberrations are able to escape apoptosis and give rise to CIN.

The oncogenic activity of GLI2 interacts with a number of biological processes, leading to the inhibition of apoptosis, resistance to growth arrest and suppression of epithelial differentiation; as such, Gli2ΔN alone is sufficient for the generation of BCC tumors.^17, 18, 19^ In this study we show for the first time, to our knowledge, that activation of GLI2ΔN can induce GIN, a widespread feature of human cancer. There are two mechanisms that cause GIN: (a) the increased genomic alteration rate and (b) the loss of the surveillance of cells with genomic defects, leading to the accumulation of cells with genomic abnormalities. Our data suggest that GLI2 interferes with both processes to contribute to GIN. By suppressing the expression of both p21^WAF1/CIP1^ and 14-3-3*σ*, GLI2 stimulates CIN by increasing the rate of bi-/multinucleated cell generation. This, along with GLI2-stimulated BCL-2 overexpression, suppress checkpoint and apoptosis activation in response to endogenous (numerical and structural chromosomal alterations) and exogenous (UVB) DNA damage. Together, GLI2 promotes CIN and the accumulation of chromosomal abnormalities, which drive tumor formation. Although GIN is a hallmark of human cancer, we provide evidence, indicating that GLI2 may have a key role in driving such events in human neoplasms.

## Material and Methods

### Plasmids and cloning

The self-inactivating retroviral vector (pSIN)-MCS vector, containing an MCS insert, was cloned from pSIN-CMV-EGFP plasmid.^63^ The pSIN-CMV-GLI2ΔN-stable expression retroviral transduction vector was obtained by excising the CMV-EGFP-GLI2ΔN ORF from pCMV-EGFP-GLI2ΔN with *Sal*I and *Mfe*I and subcloning it into the pSIN-MCS vector cut with *Xho*I and *Eco*RI.

### Cell lines and culture

Human telomerase reverse-transcriptase (h/TERT)-immortalised N/TERT-1 are derived from clinically normal foreskin tissue and were supplied by Professor James Rheinwald (Department of Dermatology, Harvard University Medical School, Boston, MA, USA). The telomerase-immortalised cell line, N/TERT-1 (N/TERT)^64^ was chosen to examine GLI2ΔN effects because of its genotype, which is comparable to primary human keratinocytes.^64^ This line retains a functional p53/p21^WAF1/CIP1^ pathway, and although it has a potentially unlimited lifespan because of abrogated senescence, it is neither tumour derived nor malignant.^64^ N/TERT keratinocytes were cultured in Dulbecco's modified Eagle's medium (DMEM) high glucose, supplemented with 25% (v/v) Ham's F12 medium (PAA, Somerset, UK), 10% (v/v) fetal calf serum (FCS; Biosera, East Sussex, UK), 1% (v/v) Glutamine (PAA), 1% (v/v) penicillin/streptomycin (PAA) and various mitogens (0.4 *μ*g/ml hydrocortisone (Sigma-Aldrich, Dorset, UK), 0.1 nM cholera toxin (BioMol, Exeter, UK), 5 *μ*g/ml transferrin (Sigma-Aldrich), 20 pM liothyronine (Sigma-Aldrich), 5 *μ*g/ml insulin (Sigma-Aldrich) and 10 ng/ml epidermal growth factor (AbD Serotec, Kidlington, UK)). Pimary NHEKs were cultured in a low CaCl_2_ (0.1 mM; Gibco, Paisley, UK) keratinocyte serum-free medium (K-SFM; Life Technologies, Paisley, UK), supplemented with 0.3 mM CaCl_2_ (Sigma-Aldrich), 25 *μ*g/ml bovine pituitary extract (Life Technologies), 0.2 ng/ml human epidermal growth factor (Life Technologies) and 1% (v/v) penicillin/streptomycin (PAA). Diploid SK-UT-1B cells were cultured in DMEM (PAA) high glucose, supplemented with 10% (v/v) FCS, 1% (v/v) penicillin/streptomycin and 1% (v/v) glutamine. Phoenix (human embryonic epithelial 293T derived) cells supplied by Nolan Lab (Medical Center, Stanford University Medical School, Stanford, CA, USA), were cultured in DMEM supplemented with 10% (v/v) FCS, 1% (v/v) penicillin/streptomycin and 1% (v/v) glutamine. All cells were grown at 37 °C in a humidified atmosphere of either 5% (v/v) CO_2_/95% (v/v) air (for NHEK and SK-UT-1B) or 10% (v/v) CO_2_/90% (v/v) air (for N/TERT).

### Retroviral infection

All transductions using pSIN-based constructs involved transfecting the plasmid into the Phoenix packaging cell line using Fugene 6 Transfection Reagent (Roche Diagnostics, Burgess Hill, UK), according to the manufacturer's instructions, followed by selection of the cells with 3 *μ*g/ml puromycin (Sigma-Aldrich) for 7–10 days. Next, cells were incubated in normal growth medium at 32 ^°^C overnight. The supernatant from virus-containing Phoenix cells was collected once daily, one to two times post confluence. To infect N/TERT keratinocytes, NHEK and SK-UT-1B cells were pre-incubated for 10 min at 37 °C in normal growth medium containing 5 *μ*g/ml polybrene (Sigma-Aldrich), before replacing with retroviral supernatant containing the same concentration of polybrene (Sigma-Aldrich). Cells were centrifuged (350 × *g*) at 32 °C for 1 h before retroviral supernatant was replaced with normal growth medium and were kept in normal culture condition. Transduced cells were incubated for at least 24 h before being used for experiments. Because of lack of selectable markers, transduced cells were fluorescent-activated cell sorted and collected in order to enhance the percentage of cells that have the retroviral construct.

### Fluorescent-activated cell sorting

All fluorescent activated cell sorting (FACS) runs (sorting based on enhanced green fluorescence protein – EGFP levels) were performed in a BD FACSAria Cell Sorter fitted with a Blue Argon Laser 488 nm, violet diode 405 nm and red diode 633 nm (BD Biosciences, San Jose, CA, USA). After washing with 1 × PBS, cell pellets were resuspended in 1 × PBS each and then filtered through a 70-*μ*m strainer. N/TERT and NHEK and SK-UT-1B wild-type cells were used for obtaining basal levels of green fluorescence (background autofluorescence), and all cells above this basal threshold were considered as being EGFP-positive fluorescent cells, and were thus sorted and plated in normal growth medium.

### Light and fluorescence microscopy

Normal cultured cells, cultured cells expressing EGFP or EGFP-GLI2ΔN fusion protein were visualised using a light fluorescence microscope (Leica DM IRB/Nicon Eclipse TE-2000-S, Leica Microsystems, Milton Keynes, UK) to detect cell morphology and gene/protein expression. Images were captured with a digital imaging system (Leica DC2000 camera) attached to the microscope.

### CAsy cell counter

Cell number and density of viable cells were determined using CAsy Cell Counter (Innovatis, Sittingbourne, UK). Each sample (cell suspension) was prepared three times in CAsyTon (Innovatis) buffer, followed by triplicate measurements of 200 *μ*l sample volume. Viable cells were measured by Casy Cell Counter, by excluding all counts that were of a size smaller that 10 *μ*m (dead cells and debris).

### Proliferation assays

For MTT proliferation assay, cells were seeded in separate (one for each time point) 24-well plates. The next day, cells were incubated for 2 h in MTT (Sigma-Aldrich) and solution was prepared (growth medium containing 10% (v/v) of MTT stock solution 5 mg/ml) at 37 °C in a humidified atmosphere of 10% (v/v) CO_2_/90% (v/v) air (Day 0). MTT formazan crystals were solubilised in isopropanol (Fisher, Leicestershire, UK) and 100 *μ*l of the solution from each well was transferred to a well of a clear, flat-bottomed 96-well plate (Nunc, Roskilde, Denmark). The absorbance (optical density) was measured at 560 nm, using a 96-well microplate reader (FLUOSTAR OPTIMA microplate reader, BMG LABTECH, Aylesbury, UK). For Alamar Blue assay, cells were incubated in 10% Alamar Blue (Invitrogen, Paisley, UK) solution in growth medium for 6 h at 37 °C in a humidified atmosphere of 10% (v/v) CO_2_/90% (v/v) air (Day 0). Following incubation, 100 *μ*l of the target cell medium was obtained and added in each well of an opaque, flat-bottomed 96-well microtiter white plate (Nunc). Alamar Blue (Invitrogen) reduction (fluorescence) was read on a FLUOSTAR OPTIMA microplate reader (BMG LABTECH) at 544 excitation wavelength and at 590 nm emission wavelength. Next, medium containing the Alamar Blue (Invitrogen) was aspirated and cells were re-fed with fresh normal growth medium. Samples were analysed in six replicates.

### Population doublings

Keratinocyte population doublings were calculated as follows: PD=3.32 × (log_10_ (N1)−log_10_ (N0)), where N1=total yield (total output) and N0=initial number of seeded keratinocytes. All PD values were individually collected (once every 4 days) and were used cumulatively.

### Flow cytometry analysis

All flow cytometry analytical runs were performed on a BD LSRII fitted with a Blue Argon Laser 488 nm, violet diode 405 nm, red diode 633 nm and a UV laser 325 nm (BD Biosciences), using the BD FACSDiva Software (BD Biosciences).

For flow cytometry-Hoechst-33342 (Sigma-Aldrich) analysis, cells were trypsinised and centrifuged at 1000 r.p.m. for 5 min. After washing with 1 × PBS and spinning at 1000 r.p.m. for 5 min, cell pellets were resuspended in Hoechst-33342 10 *μ*g/ml in 1 × PBS. After 1-h incubation at 37 °C in a humidified atmosphere of 10% (v/v) CO_2_/90% (v/v) air, cells were filtered through a 70-*μ*m strainer and cellular DNA content was measured by flow cytometry.

For flow cytometry—propidium iodide (Sigma-Aldrich) analysis, culture medium was centrifuged together with trypsinised cells to collect all cells, including detached cells, at 1500 r.p.m. for 5 min. After washing with 1 × PBS and spinning at 1500 r.p.m. for 5 min, cell pellets were resuspended in ice-cold 70% (v/v) ethanol and incubated for 2 h at 4 °C. Cells were then centrifuged at 6000 r.p.m. for 2 min. Supernatant was aspirated and each pellet was washed in 1 × PBS and centrifuged at 6000 r.p.m. for 2 min. Cell pellets were then resuspended in 100 mM NaCitrate (Sigma-Aldrich) each and centrifuged at 6000 r.p.m. for 2 min. Next, supernatant was aspirated and each pellet was resuspended in 300–500 *μ*l PI/RNaseA mixture (50 *μ*g/ml propidium iodide, 125 *μ*g/ml RNase A, 38 mM NaCitrate, 1 × PBS; all supplied by Sigma-Aldrich). Resuspended cells were incubated for 10–20 min in the dark at room temperature and cellular DNA content measurement by flow cytometry was carried out, where the same number of events was acquired for each sample (25 000–50 000 events). Samples were analysed either in duplicates or triplicates.

### Cy5 Annexin V staining

For Cy5 Annexin V (BD Biosciences) and DAPI staining followed by flow cytometry analysis, equal numbers of N/TERT, SINCE and SINEG2 cells were seeded in 6-cm dishes, and collected either 24 or 48 h after plating, or were collected after 24 h of mock treatment or UVB treatment for 24 h in order to detect UVB-induced apoptosis. Culture media from each of UVB-treated culture dishes were also collected to include any detached dead or apoptotic cells that may be floating in the medium. After centrifugation, cells were resuspended in 100 *μ*l of 1 × Annexin V-binding buffer solution (BD Biosciences) and 5 *μ*l Cy5 Annexin V antibody (BD Biosciences) or without antibody (negative controls). Resuspended cells were then gently vortexed and were incubated for 15 min at room temperature in the dark. Next, 400 *μ*l of 1 × Annexin V-binding buffer solution (BD Biosciences) was added per sample and samples were kept on ice. Finally, DAPI (Sigma-Aldrich) was added at a final concentration of 200 ng/ml per sample, 1–2 min before the cells were given for flow cytometry analysis, where the same number of events was acquired for each sample (20 000–30 000 events). Samples were analysed in duplicates.

### Nuclear staining

For Hoechst-33342 staining, cells were seeded at equal densities, and as soon as they reached 50–60% confluence they were washed, trypsinised and stained with 10 *μ*g/ml Hoechst-33342 (Sigma-Aldrich) in suspension for 1 h at 37 °C. Cells were then seeded at equal densities, and digital bright-field and fluorescent images were captured 24, 48 and 72 h (∼30–60% confluent throughout the experiment) after plating.

For DAPI staining, cells were fixed 72 h after plating (∼40–60% confluent) in 4% (v/v) formaldehyde/formalin (Fisher) solution for 20 min at room temperature and were stained with 200 ng/ml DAPI (Sigma-Aldrich) for 10 min at room temperature in the dark. Digital bright-field and fluorescent images (∼15 images (fields) per cell line in two replicate wells) were captured using a fluorescence microscope. The binucleated (tetraploid) cells per field (image) were counted using Adobe Photoshop CS4 Extended (Adobe Systems Europe, Bergshire, UK). The percentage of binucleated cells per field was derived by counting the number of cells with two nuclei over the total number of cells per field. Thus, the percentage of binucleated cells per field (*x*) was calculated as follows: *x*=number of binucleate cells × 100/total number of cells. An average of ∼300–350 cells were counted for each cell line in total. Only binulceated cells were counted in this analysis, whereas all multinucleated cells were included in the total number of cells per field. Cells undergoing mitosis were also counted as two mononuclear cells.

### Reverse-transcription PCR

Total RNA was extracted from cells using RNeasy Mini Kit (Qiagen, West Sussex, UK) and RNA (1–2 *μ*g) was reverse transcribed into cDNA with the Reverse transcription kit (Promega, Hampshire, UK) according to manufacturer's protocols.

### Real-time quantitative PCR

Real-time quantitative PCR (qRT-PCR) was performed as previously described.^65^ Briefly, qRT-PCR runs were performed in the LightCycler 480 qPCR system (Roche Diagnostics). Relative quantification of mRNA transcripts was performed by LightCycler 480 Advanced Relative Quantification software (Roche Diagnostics) with at least two reference genes for each analytical run. Samples were analysed in triplicates and significance values were calculated by Graphpad Instat software (GraphPad Software, San Diego, CA, USA) by using two-sided *P*-values or using ANOVA analysis for multiple sample comparisons. All primer sequences are listed in the Supplementary Table S1. Using the GeNorm algorithm analysis method,^66^ and the LightCycler 480 Relative Quantification Software programme (Roche Diagnostics; with built-in multiple reference genes normalisation algorithm), of the eight reference genes (*GAPDH*, *RPLPO*, *YAP1*, *UBC*, *HPRT1*, *POLR2A*, *ESD* and *18S*), two were identified as being most reliable and stable reference genes: polymerase (RNA) II (DNA directed) polypeptide A (*POLR2A*) and Yes-associated protein 1 (*YAP1*) across a large panel of normal and cancer human cells and tissues.^65^

### Semiquantitative PCR

Semiquantitative PCR analysis was performed in Verity 96-well Thermal Cycler (Applied Biosystems, Foster City, CA, USA) for the visualisation of *GLI2* (whole-gene amplification or *α*/*β*-isoform-specific amplification) gene mRNA expression. Samples were run on a typical PCR amplification protocol (95 °C for 10 s, 60 °C for 6 s, 72 °C for 6 s) for 36 cycles for *GLI2* full-length and *POLR2A*, and to 27 and 30 cycles for *GLI2* a/*β* and *POLR2A*, respectively. PCR products were visualised by UV light and a gel image was captured in the Autochemi imaging system (UVP). Primer sequences are listed in Supplementary Table S1.

### Immunoblot analysis

Total cell protein extraction and immunoblot analysis was performed as previously described.^65^ For the extraction of total protein from UVB-irradiated cells, culture media were also collected by centrifugation to include any dead or apoptotic cells in the medium. Primary antibodies used were: goat polyclonal anti-14-3-3*σ* (N-14, sc-7681, Santa Cruz, Heidelberg, Germany), mouse monoclonal anti-Bcl-2 (100, sc-509, Santa Cruz), rabbit polyclonal anti-Caspase-3 (Cell Signaling, Danvers, MA, USA), rabbit polyclonal anti-EGFP (ab-290, Abcam, Cambridge, UK), rabbit polyclonal anti-GLI2 (H300, Santa Cruz), mouse monoclonal anti-p21^WAF1/CIP1^ (Santa Cruz), mouse monoclonal anti-p53 (DO1, CRUK, Lincoln's Inn Fields, London, UK) and mouse monoclonal anti-*β*-actin, Sigma-Aldrich). Secondary HRP antibodies were as follows: polyclonal rabbit anti-mouse immunoglobulin/HRP (DakoCytomation, Cambridgeshire, UK), polyclonal goat anti-rabbit immunoglobulin/HRP (DakoCytomation) and polyclonal rabbit anti-goat immunoglobulin/HRP (DakoCytomation).

### UVB irradiation

Initially, the growth medium of semiconfluent (50–65%) cells was aspirated. The culture dishes were then inserted in the UV cross-linker (UVP) and the lids were removed. Cells were irradiated with UVB at different doses (10–30 mJ/cm^2^) and fresh growth medium was replaced immediately after UVB exposure. Routine calibration was performed to ensure UVB emission peak at 312 nm, which is physiologically relevant to skin photobiology. Similar procedure was followed for the control cells except the UVB-exposure step (non-UVB-treated control cells).

### Navitoclax (ABT-263) treatment

Cells were grown in monolayer until ∼60% confluence. Next, cells were treated with a range of different doses of Navitoclax (ABT-263) dissolved in DMSO. Untreated cells were always used as controls and exposed to equal volume of DMSO as for treated cells. Navitoclax (ABT-263) was purchased from Selleck Chemicals (Houston, TX, USA).

### Multiplex fluorescent *in situ* hybridisation

Metaphase cells were collected by standard cytogenetic methods. Twenty four-colour FISH was carried out as previously described^67, 68^ by following the Vysis M-FISH protocol and using the SpectraVysion DNA probe (Abbott Molecular, Berkshire, UK). The computer software SpectroVision (Abbott Molecular) was used to capture and analyse the images. More than 10 M-FISH karyotypes were analysed for each cell line and rearrangements detected in two or more metaphases were considered as clonal.

### Immunohistochemistry analysis of BCC clinical samples

The protein expression levels of p21^WAF1/CIP1^ (p21) were examined in 16 biopsies comprising normal skin and human BCC subtypes, including nodular (*n*=9), superficial (*n*=4) and infiltrative (*n*=3). Immunohistochemistry analysis of p21^WAF1/CIP1^ was performed using p21^WAF1/CIP1^ mouse monoclonal antibody (DCS60, Cell Signaling) at 1 : 125 dilution. Tissue processing and tissue sections were cut at the Pathology Department of the Institute of Cancer, Barts and the London School of Medicine and Dentistry, Charterhouse Square, London, UK. Briefly, 4-*μ*m tissue sections were cut from the BCC paraffin blocks and transferred on to charged microscope slides. The slides were dried in a 37-°C oven overnight and then run on Ventana discovery platform (Ventana Molecular Discovery Systems, Roche Diagnostics) with an automated protocol, including deparaffinisation, primary and secondary antibody incubation, DAB application, haematoxylin and bluing reagent counterstaining and finally slides cleaning. After the run was completed, the slides were placed in soapy tap water and rinsed under the tap for 1 min and then placed in dH_2_O. Following dehydration in a series of solvents, the slides were mounted with coverslips.

### Affymetrix 10 K SNP analysis

Genomic DNA samples were purified and processed as described previously.^65^ Briefly, 350–500 ng of DNA was digested with *Xba*I and ligated to the *Xba*I adaptor before PCR amplification using AmpliTaq Gold with Buffer II (Applied Biosystems). The PCR amplification profile on an MJ DNA Engine thermal cycler was: 95/180 (hot start) and 95/20, 59/15, 72/60 (°C/s) for 40 cycles followed by a termination step at 72 °C for 7 min. PCR products were purified using Ultrafree-MC 30 000 NMWL filter columns (catalogue number UFC-3LTK00; Millipore, Watford, UK) and DNA concentrations determined using NanoDrop spectrophotometer. Hybridisation and scanning of the arrays were processed as described previously.^65^ Evaluation of genomic copy number alterations was performed using Affymetrix Copy Number Analysis Tool software (CNAT, version 4) where the threshold for statistical significance was *P*≤10^−6^ as recommended by Affymetrix. The data was cross verified and genome ploidy ±S.D. for Log2 ratio values were obtained using an independent algorithm, Copy Number Analyzer for GeneChip (version 2; Department of Regeneration Medicine, University of Tokyo, Tokyo, Japan). Average SNP call rates for all chips were 95–99%.

### Statistical analysis

Statistical analysis was performed using either the Microsoft Excel's software or the GraphPad's InStat (V2-04a) and Prism Software (V5,0; GraphPad Software) for Student's *t*-test analysis.

## Abbreviations

BCC: basal cell carcinoma
BCL-2: B-cell lymphoma 2
CIN: chromosomal instability
EGFP: enhanced green fluorescent protein
FACS: fluorescence-activated cell sorting
GIN: genomic instability
GLI1: glioma 1
GLI2: glioma 2
GLI2ΔN: GLI2-*β* isoform, which lacks the N-terminal repressor domain
h/TERT: human telomerase reverse transcriptase
HH: Hedgehog
M-FISH: multiplex fluorescent *in situ* hybridisation
MTT: 3-(4,5-dimethylthiazol-2-yl)-2,5-diphenyl tetrazolium bromide
N/TERT: human telomerase-immortalised newborn epidermal keratinocyte
NHEK: normal human epidermal keratinocyte
pSIN: self-inactivating retroviral vector
SINCE: N/TERT keratinocytes stably expressing EGFP
SINEG2: N/TERT keratinocytes stably expressing EGFP-GLI2ΔN
SK-UT-1B: human uterus endometrium leiomyosarcoma
SNP: single nucleotide polymorphism
