# Supplementary material for: GLI2 induces genomic instability in human keratinocytes by inhibiting apoptosis
Source: Cell Death Dis. 2014 Jan 30;5(1):e1028–. doi: 10.1038/cddis.2013.535 (PMC4040660; doi:10.1038/cddis.2013.535)
Supplement: Supplementary Figures [file cddis2013535x1.pdf]

Figure S1

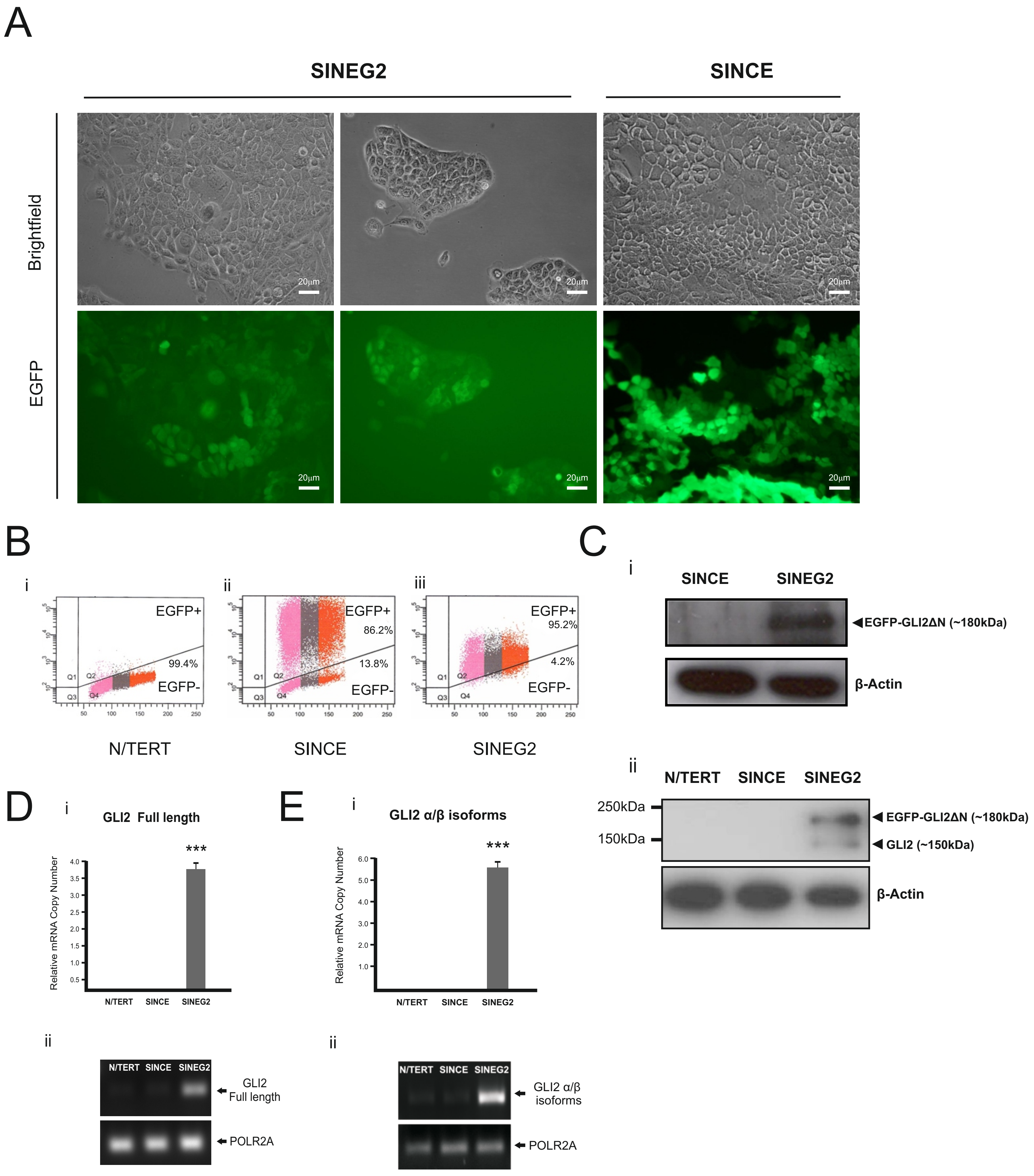

A
SINEG2
SINCE
Brightfield
EGFP
20µm
B
i
ii
iii
EGFP+
EGFP-
99.4%
86.2%
13.8%
95.2%
4.2%
Q1
Q2
Q3
Q4
N/TERT
SINCE
SINEG2
C
i
SINCE
SINEG2
◀EGFP-GLI2ΔN (~180kDa)
β-Actin
ii
N/TERT
SINCE
SINEG2
250kDa
150kDa
◀ EGFP-GLI2ΔN (~180kDa)
◀ GLI2 (~150kDa)
β-Actin
D
i
GLI2 Full length
Relative mRNA Copy Number
***
N/TERT
SINCE
SINEG2
ii
GLI2
Full length
POLR2A
E
i
GLI2 α/β isoforms
Relative mRNA Copy Number
***
N/TERT
SINCE
SINEG2
ii
GLI2 α/β
isoforms
POLR2A

Figure S2

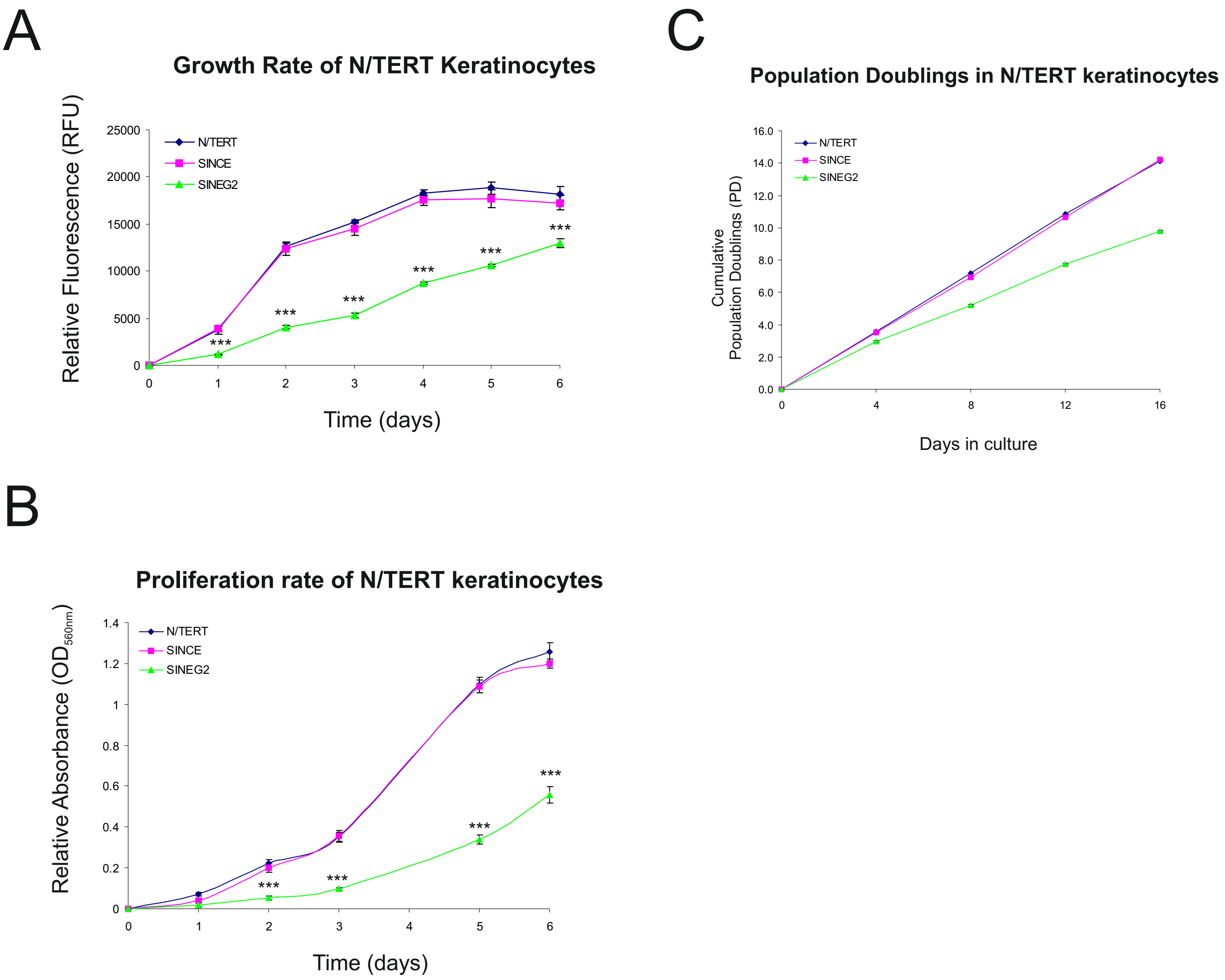

A
Growth Rate of N/TERT Keratinocytes
Relative Fluorescence (RFU)
25000
20000
15000
10000
5000
0
N/TERT
SINCE
SINEG2
***
0
1
2
3
4
5
6
Time (days)
C
Population Doublings in N/TERT keratinocytes
Cumulative Population Doublings (PD)
16.0
14.0
12.0
10.0
8.0
6.0
4.0
2.0
0.0
N/TERT
SINCE
SINEG2
0
4
8
12
16
Days in culture
B
Proliferation rate of N/TERT keratinocytes
Relative Absorbance (OD560nm)
1.4
1.2
1
0.8
0.6
0.4
0.2
0
N/TERT
SINCE
SINEG2
***
0
1
2
3
4
5
6
Time (days)

Figure S3

A

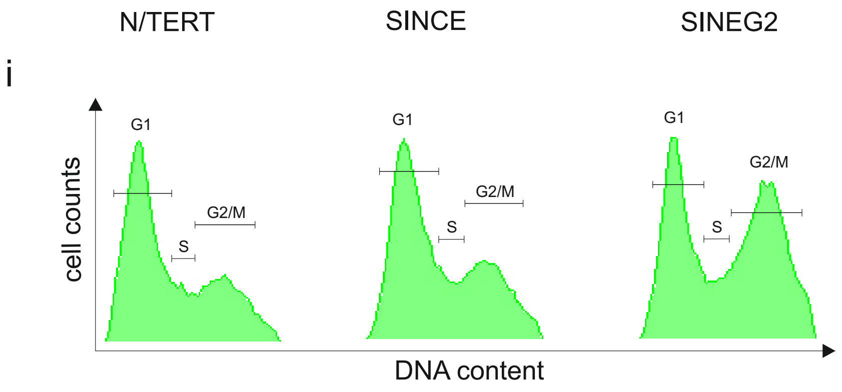

ii

	G1	S	G2/M
N/TERT	54.8% ± 2.6	19.7% ± 2.9	26.3% ± 1.8
SINCE	51.8% ± 1.8	21.3% ± 2.1	28.1% ± 0.8
SINEG2	42.1% ± 1.1	17.8% ± 1.8	41.9% ± 2.1

B

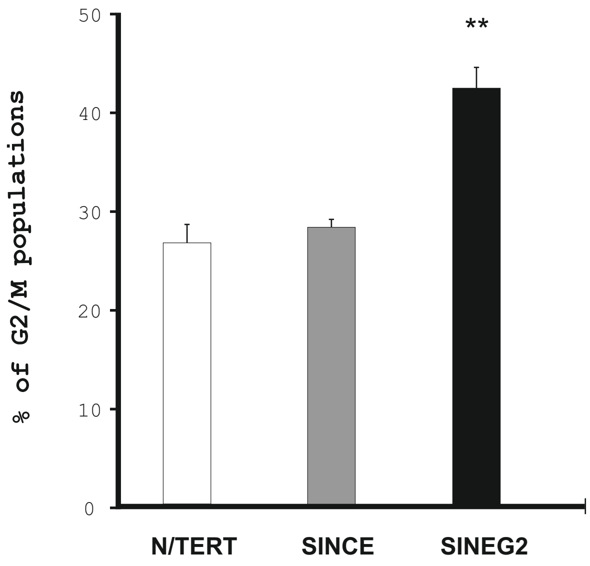

Figure S4

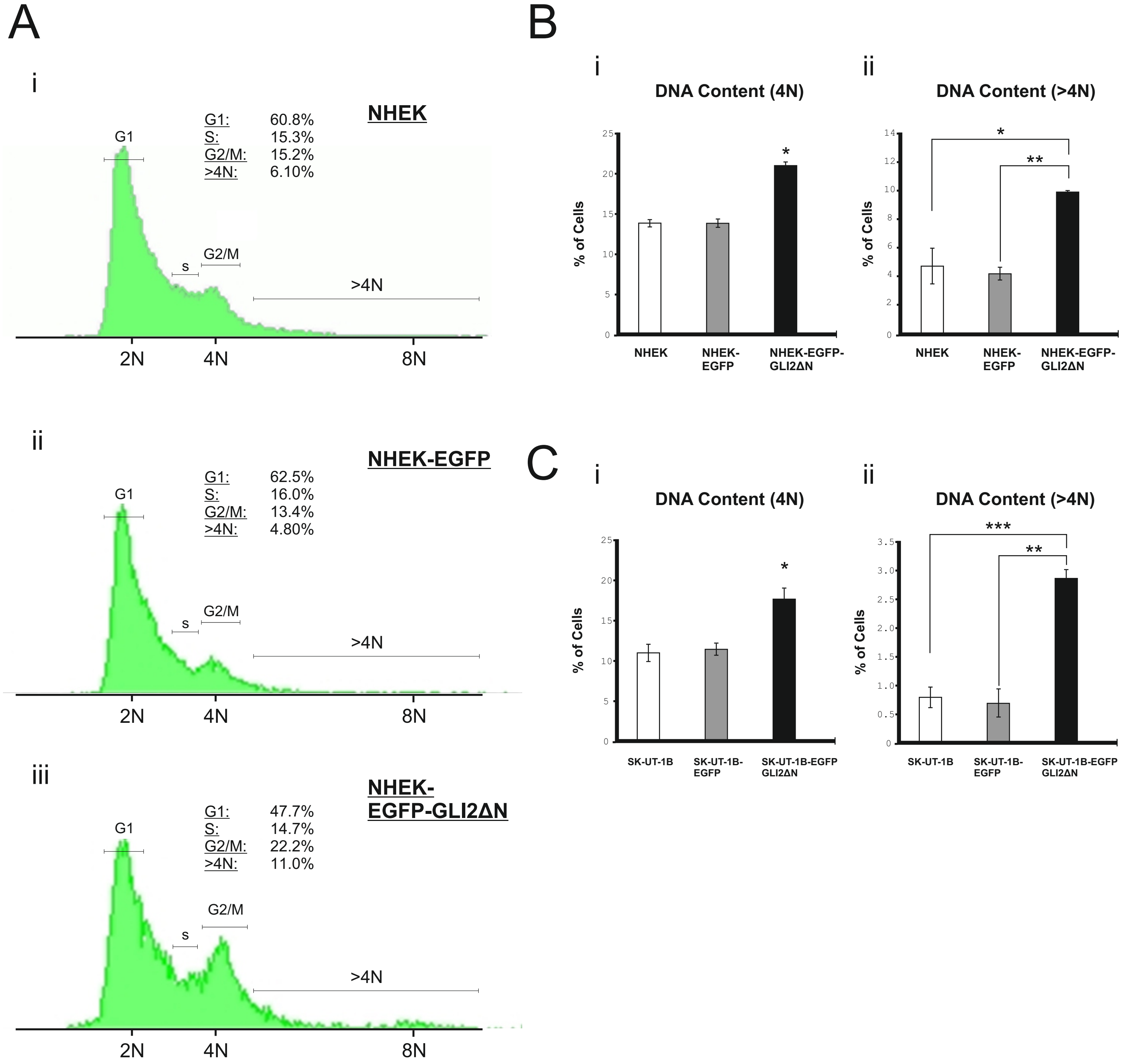

A
i
NHEK
G1: 60.8%
S: 15.3%
G2/M: 15.2%
>4N: 6.10%
G1
s
G2/M
>4N
2N
4N
8N
ii
NHEK-EGFP
G1: 62.5%
S: 16.0%
G2/M: 13.4%
>4N: 4.80%
iii
NHEK-EGFP-GLI2ΔN
G1: 47.7%
S: 14.7%
G2/M: 22.2%
>4N: 11.0%
B
i
DNA Content (4N)
% of Cells
NHEK
NHEK-EGFP
NHEK-EGFP-GLI2ΔN
ii
DNA Content (>4N)
C
i
DNA Content (4N)
SK-UT-1B
SK-UT-1B-EGFP
SK-UT-1B-EGFP GLI2ΔN
ii
DNA Content (>4N)

Figure S5

A

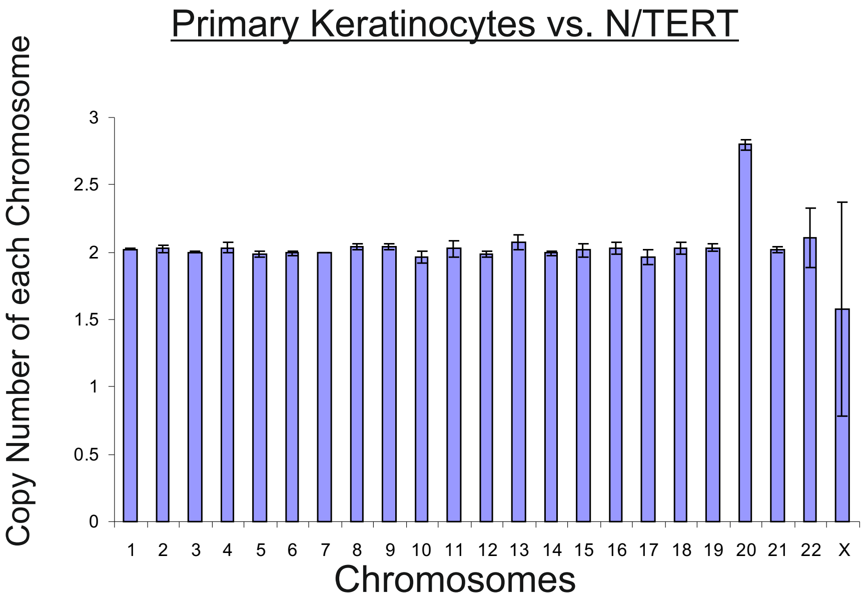
Primary Keratinocytes vs. N/TERT
Copy Number of each Chromosome
3
2.5
2
1.5
1
0.5
0
1 2 3 4 5 6 7 8 9 10 11 12 13 14 15 16 17 18 19 20 21 22 X
Chromosomes

B

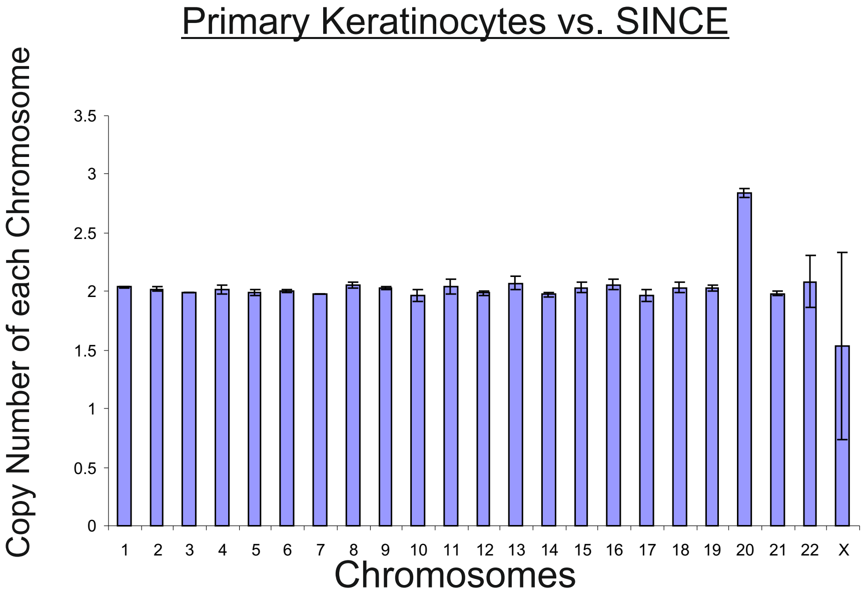
Primary Keratinocytes vs. SINCE
Copy Number of each Chromosome
3.5
3
2.5
2
1.5
1
0.5
0
1 2 3 4 5 6 7 8 9 10 11 12 13 14 15 16 17 18 19 20 21 22 X
Chromosomes

Figure S6

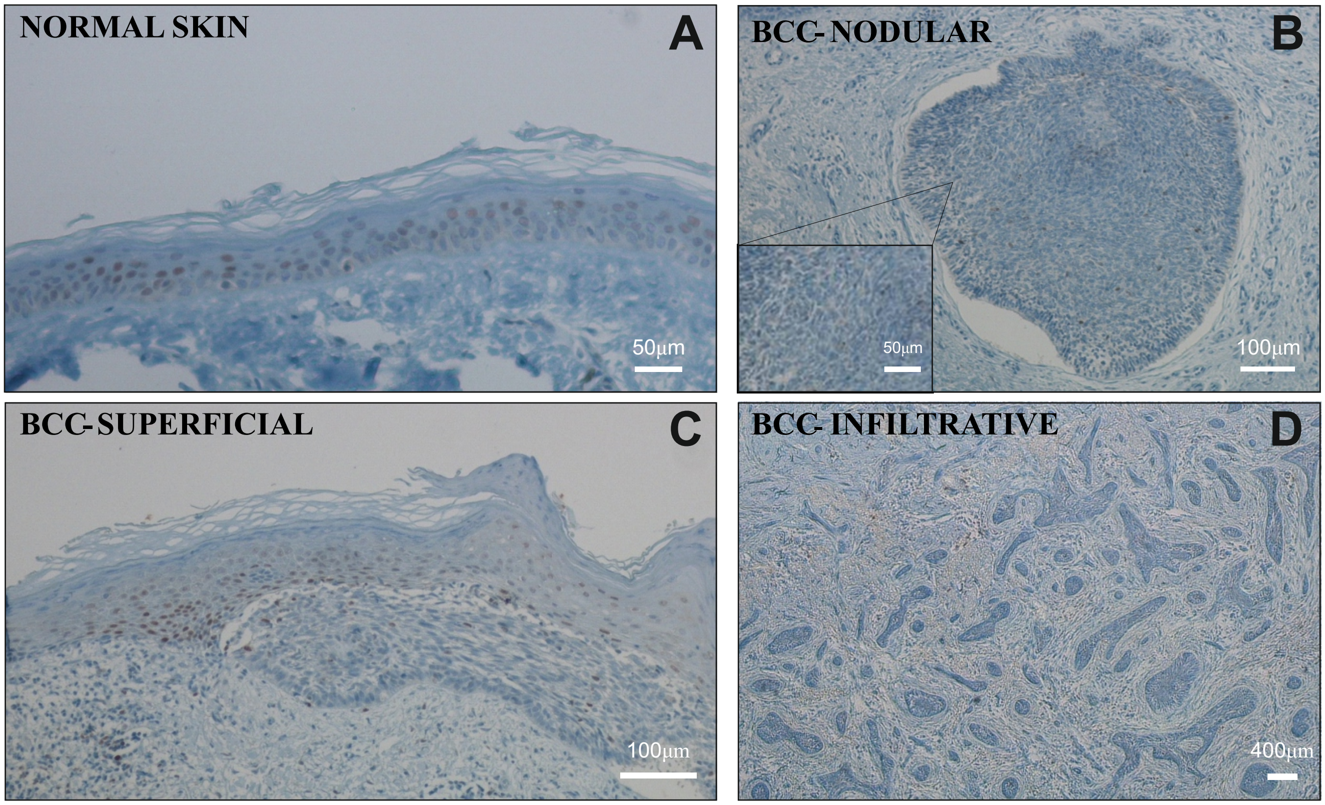
NORMAL SKIN
A
50μm
BCC- NODULAR
B
50μm
100μm
BCC- SUPERFICIAL
C
100μm
BCC- INFILTRATIVE
D
400μm

Figure S7

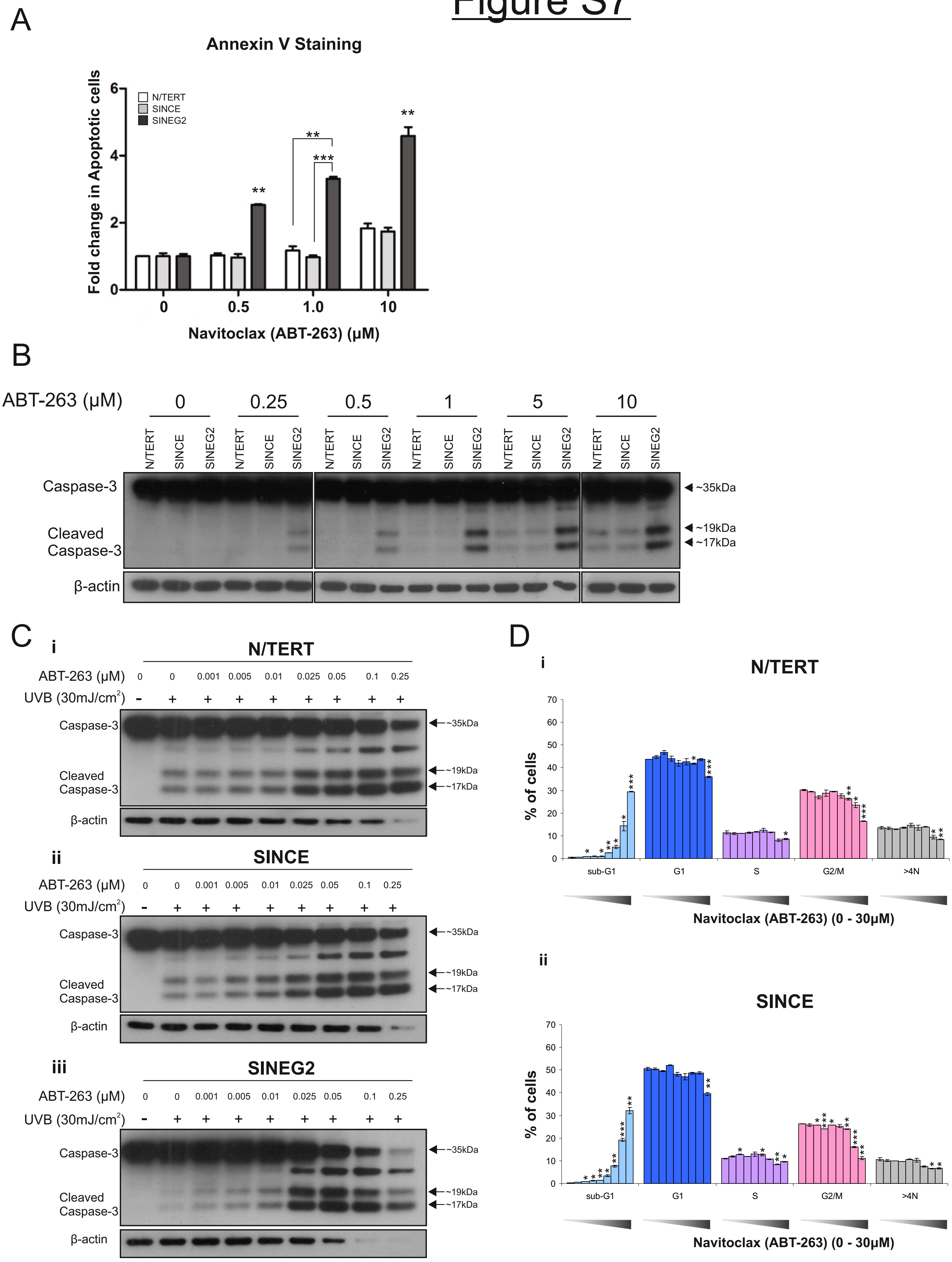
A
Annexin V Staining
Fold change in Apoptotic cells
N/TERT
SINCE
SINEG2
Navitoclax (ABT-263) (µM)
B
ABT-263 (µM)
0
0.25
0.5
1
5
10
Caspase-3
Cleaved Caspase-3
β-actin
~35kDa
~19kDa
~17kDa
C
i N/TERT
ii SINCE
iii SINEG2
ABT-263 (µM)
UVB (30mJ/cm²)
D
i N/TERT
ii SINCE
% of cells
sub-G1
G1
S
G2/M
>4N
Navitoclax (ABT-263) (0 - 30µM)
