# Supplementary material for: GLI2 induces genomic instability in human keratinocytes by inhibiting apoptosis
Source: Cell Death Dis. 2014 Jan 30;5(1):e1028–. doi: 10.1038/cddis.2013.535 (PMC4040660; doi:10.1038/cddis.2013.535)
Supplement: Supplementary Figure Legends [file cddis2013535x2.doc]

**Supplementary Figure Legends**

Figure S1: Generation of stable EGFP-GLI2ΔN expressing keratinocytes.

**(A)** Representative pictures of EGFP-GLI2ΔN fusion protein and EGFP stably expressing N/TERT cells (x20) under brightfield and UV excitation for EGFP fluorescence, showing clear expression of EGFP-GLI2ΔN (SINEG2) and EGFP (SINCE) proteins. The EGFP-GLI2ΔN fusion protein localisation is mainly nuclear and partly cytoplasmic. **(B)** Flow cytometry analysis of wild-type N/TERT cells **(i)**, used as reference cell line, against both SINCE **(ii)** and SINEG2 **(iii)** cell line, showing the presence of distinct populations of EGFP expressing cells. **(C)** **(i)** Immunoblotting of SINCE and SINEG2 cell protein extracts against anti-EGFP antibody revealed the presence of a band at the predicted size of EGFP-GLI2ΔN fusion protein at ~ 180 kDa. **(ii)** Immunoblotting of N/TERT, SINCE and SINEG2 cell protein extracts against anti-GLI2 antibody revealed the presence of a band at the predicted size of EGFP-GLI2ΔN exogenous fusion protein at ~ 180 kDa and a band at the predicted size of GLI2 endogenous protein at ~ 150 kDa in SINEG2 cells. β-actin (~ 42 kDa) was used as loading control. **(D)** RNA was harvested from N/TERT, SINCE, and SINEG2 cells to examine the levels of GLI2 expression using primers specific for full length GLI2. Quantitative RT-PCR analysis of GLI2 full length expression **(i)** and semi-quantitative PCR **(ii)** analysis, show the presence of high levels of GLI2 mRNA expression in SINEG2 cells compared to N/TERT and SINCE, cells where GLI2 mRNA expression is low but detectable. Each bar represents a mean ± s.e.m of triplicate samples. **** P≤0.001*. **(E)** RNA was harvested from N/TERT, SINCE, and SINEG2 cells to examine the levels of GLI2 expression using primers to amplify α/β isoforms of GLI2. Quantitative RT-PCR analysis of GLI2 α/β isoforms expression **(i)** and semi-quantitative PCR **(ii)** analysis show the presence of high GLI2 α/β expression in SINEG2 cells compared to N/TERT and SINCE cells where GLI2 α/β mRNA expression is low but detectable. Each bar represents a mean ± s.e.m of triplicate samples. **** P≤0.001*. POLR2A was used as a housekeeping gene control for all samples in semi-quantitative PCR.

Figure S2: GLI2ΔN overexpression reduces the growth rate of N/TERT keratinocytes.

**(A)** N/TERT, SINCE, and SINEG2 keratinocytes were grown for a period of seven days and their growth rates were measured by the fluorescence intensity produced by Alamar Blue^TM^ reduction. SINEG2 display a significantly reduced growth rate compared to both N/TERT and SINCE control keratinocytes. **(B)** The proliferation rate of keratinocytes was also assayed by the rate of MTT reduction during the period of seven days in culture. Again, GLI2ΔN expressing N/TERT keratinocytes display a significantly slower growth rate compared to both N/TERT and SINCE control keratinocytes. Each data point in the graphs represents a mean ± s.e.m of six replicate samples, relative to the baseline values obtained at Day 0. All the values for all the different time points were subtracted from the mean value of Day 0. **** P≤0.001*.**(C)**N/TERT, SINCE, and SINEG2 cells were serially passaged every 4 days for a total of 16 consecutive days. GLI2ΔN expressing N/TERT keratinocytes have undergone significantly less population doublings after 16 days in culture compared to the N/TERT and SINCE cells. Each data point in the graph represents the PD which was calculated by using the mean ± s.e.m of three replicate measurements, as described in materials and methods.**** P≤0.001*.

Figure S3: Stable expression of GLI2ΔN increases the 4N population of N/TERT cells.

**(A)** Flow cytometry analysis of stable EGFP-GLI2ΔN cell line (SINEG2) against both N/TERT and SINCE control cells after Hoechst-33342 staining **(i)**. After doublet discrimination with width and area parameters, cells were discriminated according to DNA content and the cell cycle profile was obtained. A significant increase in the cell population at the G2/M boundary is evidenced in the SINEG2 cell line, compared to both N/TERT and SINCE cells. Average values of three independent experiments are presented **(ii)**. **(B)** Graphical presentation of the percentage of G2/M populations, to indicate the significant differences in the presence or absence of EGFP-GLI2ΔN expression. Each bar represents the mean values ± s.e.m of three independent experiments. ** *P≤0.01*.

Figure S4: GLI2ΔN increases the 4N and >4N populations in primary normal human epidermal keratinocytes (NHEK) and SK-UT-1B diploid cancer cells.

**(A)** Hoechst-33342 staining, followed by flow cytometry analysis to obtain cell cycle distribution of wild-type NHEK **(i)**, and NHEK transduced with either EGFP, (NHEK-EGFP) **(ii)**, or EGFP-GLI2ΔN, (NHEK-EGFP-GLI2ΔN) **(iii)** cells. Data are representative of two independent experiments including two independent donor samples. **(B)** Graphical representation of the percentage of **(i)** 4N and **(ii)** >4N cells, for each cell line after Hoechst-33342 staining and flow cytometry analysis. NHEK-EGFP-GLI2ΔN cells have significantly higher percentage of tetraploid/near tetraploid and aneuploid cells in culture. Each bar represents the mean values ± s.e.m of two independent donor samples. **(C)** Graphical representation of the percentage of **(i)** 4N and **(ii)** >4N cells, for parental SK-UT-1B and SK-UT-1B transduced with either EGFP (SK-UT-1B-EGFP), or EGFP-GLI2ΔN (SK-UT-1B-EGFP-GLI2ΔN) cells after Hoechst-33342 staining and flow cytometry analysis. SK-UT-1B-EGFP-GLI2ΔN cells have significantly higher percentage of tetraploid/near tetraploid and aneuploid cells in culture. Each bar represents the mean values ± s.e.m of three independent experiments. **N**: haploid number. ** P≤0.05*, *** P≤0.01*, **** P≤0.001*.

Figure S5: 10K SNP Microarray Mapping Assay of human N/TERT keratinocytes.

The ploidy status of N/TERT keratinocytes was examined by means of 10K SNP Microarray Mapping Assay (GEO Accession Number: GSE36105). Genomic DNA from N/TERT (top panel) and SINCE (bottom panel) keratinocytes was analysed against reference genomic DNA of primary keratinocytes derived form two separate healthy donors. The average ploidy status of all the SNPs located on each chromosome after Affymetrix GTYPE analysis was plotted. Both N/TERT and SINCE keratinocytes show a normal genotype apart from the fact that chromosome 20 (trisomy) was consistently gained in all samples tested. Note that that the large error bar for chromosome X is due to the sexual variability of primary keratinocyte reference samples. Bars represent the average ploidy status of all SNPs per chromosome ± s.e.m using two individual primary keratinocyte samples as reference.

Figure S6: Reduced expression of p21^WAF1/CIP1^ in human BCCs.

Representative pictures from the immunohistochemistry for p21^WAF1/CIP1^ protein on a panel of formalin fixed, paraffin embedded **(A)** normal human skin, **(B)** nodular human BCC, **(B inset)** magnified region of nodular BCC, **(C)** superficial human BCC and **(D)** infiltrative human BCC, showing the clear downregulation of p21^WAF1/CIP1^ in human BCC tumors, compared both to normal control skin **(A)** and to normal epidermis adjacent to the BCC tumor **(C)**, with the exception of occasional positively stained cells. In normal epidermis **(A)** p21^WAF1/CIP1^ protein was detected in the nucleus of cells and was mainly observed in the differentiating suprabasal layers of the epithelium, consistent with its role in inducing cell cycle arrest prior to the onset of epithelial differentiation, with very few positive stained cells in the basal layer. Overall, these data show that the GLI2ΔN-mediated p21 downregulation *in vitro*, reflects human BCC development *in vivo*, and adds to the many other attributes shared between GLI2ΔN- overexpressing keratinocytes and BCCs (increased levels of GLI2 and Bcl-2, decreased levels of 14-3-3 sigma, ploidy abnormalities, and apoptotic resistance to UVB-mediated DNA damage ).

Figure S7: BCL-2 inhibition sensitizes GLI2ΔN-expressing cells to apoptosis and abolishes their apoptotic resistance to UVB irradiation.

**(A)** and **(B)** For direct comparison between N/TERT, SINCE, and SINEG2 cells,, in an independent experiment including fewer doses of Navitocalx, cells were either untreated (0 μM / DMSO) or treated with increasing doses of Navitoclax (ABT-263) (0.25-10 μM) for up to 24 hours and were then either analyzed by **(A)** Annexin V staining or **(B)** harvested for total cell protein and run samples from those cell lines next to each other on the same gel (Lanes 7-15 were run on a separate gel), followed by immunoblot analysis. **(A)** Staining with Cy^TM^5 Annexin V and DAPI followed by flow cytometry analysis showed that SINEG2 cells have much higher apoptotic sensitivity to BCL-2 inhibition compared to control cells. Each bar represents the mean fold change in the number of early (Annexin V (+) DAPI (-)) and late (Annexin V (+) DAPI (+)) apoptotic cells relative to the treatment with vehicle only (0 μM / DMSO) control for each individual cell line (arbitary value 1) ± s.e.m of duplicate samples. ** P≤0.05*, ***P≤0.01*, ****P≤0.001*. **(B)** Immunoblotting against anti-full length Caspase 3 (~ 35 kDa) antibody, revealed that SINEG2 cells display higher protein levels of cleaved Caspase-3 (~ 19, 17 kDa), compared to control N/TERT and SINCE cells. β-actin (~ 42 kDa) was used as a protein loading control. **(C) (i)** N/TERT, **(ii)** SINCE, and **(iii)** SINEG2 cells were either untreated (0 μM / DMSO) or treated with increasing concentrations of Navitoclax (ABT-263) (0.001 - 0.25 μM), matching those used for Annexin V staining in Fig. 5C, for up to 24 hours. Cells were either mock treated (0 mJ/cm^2^ **(-)**) or irradiated with a single dose of 30 mJ/cm^2^ **(+)** and were allowed to grow for another ~ 20 hours. Next, cells were harvested and whole cell lysates were prepared and immunoblotted against anti-full length Caspase 3 (~ 35 kDa) antibody, while β-actin (~ 42 kDa) was used as a protein loading control. Apoptosis was assessed through detecting the cleaved bands of Caspase-3 (~ 19, 17 kDa). **(D)** Cell cycle analysis of N/TERT and SINCE control cells following inhibition of BCL-2. N/TERT and SINCE cells were either untreated (0 μM / DMSO) or treated with increasing concentrations of Navitoclax (ABT-263) (0.1 - 30 μM) and were incubated for 24 hours. Next, cells were stained with propidium iodide (PI) followed by flow cytometry analysis to obtain cell cycle distribution of N/TERT and SINCE control cells. Graphical representations of flow cytometry analysis of **(i)** N/TERT and **(ii)** SINCE cells after treatment with various doses of Navitoclax (ABT-263). **(i)** Each bar represents the mean values/percentages of N/TERT cells ± s.e.m of duplicate samples present in each different phase of the cell cycle (apoptotic sub-G1, G1, S, G2/M, DNA content >4N). **(ii)** Each bar represents the mean values/percentages of SINCE cells ± s.e.m of duplicate samples present in each different phase of the cell cycle (apoptotic sub-G1, G1, S, G2/M, DNA content >4N). ** P≤0.05*, ***P≤0.01*, ****P≤0.001*. Black arrow represents the increasing doses of Navitoclax (ABT-263) (0 / DMSO, 0.1, 0.5, 1, 5, 10, 15, 20, 30 μM).
